# Association of iron deficiency and iron-deficiency anemia with behavioral problems in children with autism: a cross-sectional study

**DOI:** 10.3389/fpsyt.2026.1900958

**Published:** 2026-09-10

**Authors:** Jatan Uday Merwana, Vijay Raghunath Kalrao, Ritika Chaudhry, Leena Srivastava

**Affiliations:** Department of Pediatrics, Bharati Vidyapeeth (Deemed to be University) Medical College, Pune, Maharashtra, India

**Keywords:** autism spectrum disorder, behavioral problems, child behavior checklist, ferritin, India, iron-deficiency, iron-deficiency anemia

## Abstract

**Purpose:**

Iron deficiency (ID) and iron deficiency anemia (IDA) are common in children with autism spectrum disorder (ASD) and may contribute to behavioral problems. We examined their association with internalizing and externalizing behavioral problems.

**Methods:**

This cross-sectional study (October 2023 to March 2025) was conducted at a tertiary hospital in India. Of 179 children aged 2 to 18 years with newly diagnosed ASD (DSM-5 criteria), 173 were analyzed after six exclusions for missing data. ID and IDA were defined using age-specific World Health Organization thresholds and modeled as a three-category variable with normal iron status as the reference. The Child Behavior Checklist was used to assess internalizing and externalizing problems (T-score > 63). Multivariable logistic regression was used to estimate adjusted odds ratios (aORs), controlling for age, sex, maternal education and family type; ferritin and hemoglobin were modeled using restricted cubic splines. Bonferroni correction was applied across the six adjusted comparisons.

**Results:**

Among 173 children (median age 4.0 years [IQR 3.0–5.0]; 79.8% male), 19.7% had ID, 22.5% had IDA and 42.2% had either; internalizing and externalizing problems occurred in 39.9% and 35.8% of the children, respectively. Compared with normal iron status, both ID and IDA were associated with higher odds of internalizing problems (ID aOR 3.03, 95% confidence interval [CI] 1.34–6.86; IDA aOR 2.73, 95% CI 1.23–6.05) and externalizing problems (ID aOR 3.71, 95% CI 1.63–8.46; IDA aOR 3.24, 95% CI 1.45–7.24), corresponding to prevalence differences of 25 to 29 percentage points. An interquartile increase in ferritin (9.2 to 21.4 µg/L) was associated with lower odds of both outcomes (internalizing aOR 0.23, 95% CI 0.11–0.46; externalizing aOR 0.30, 95% CI 0.15–0.60). Five of six associations survived Bonferroni correction; IDA with internalizing problems did not. Hemoglobin was not consistently associated with either outcome.

**Conclusions:**

ID and IDA are prevalent in Indian children with ASD and are associated with clinically significant behavioral problems, with higher serum ferritin inversely associated with both outcomes. Routine iron screening may be considered during initial ASD evaluation, particularly in high-burden settings, although randomized trials are needed to determine whether correcting deficiency improves behavioral outcomes.

## 1 Introduction

Autism spectrum disorder (ASD) is a neurodevelopmental condition characterized by persistent deficits in social communication and restricted repetitive behaviors. The global prevalence is estimated at approximately 1 in 100 children (1, 2), with higher figures reported in high-income settings with intensive surveillance, such as 2.5% among children in the United States (3). Population-based estimates from five regions of India fall within the global range, at 1.0% (95% CI 0.6–1.6) in children between 2 and 6 years old and 1.4% (95% CI 0.6–3.2) in those aged 6–9 years, with variation across regions (4). Nonetheless, underdiagnosis remains common in resource-limited settings.

Approximately 58 to 70% of children with ASD have co-occurring behavioral problems, including internalizing difficulties such as anxiety and withdrawal and externalizing difficulties such as aggression and hyperactivity, which increase the caregiver burden and impair daily functioning (5–7). These behaviors often persist into adulthood, and identifying modifiable contributors is a clinical priority.

Iron-deficiency (ID) and iron-deficiency anemia (IDA) are the most prevalent micronutrient disorders worldwide, affecting approximately 40% of children aged 6–59 months (8). In India, anemia affects 67% of young children, reflecting inadequate dietary intake, infection, and malabsorption (9). Among children with ASD, the reported prevalence of ID/IDA is higher, at 24% to 52%, compared with 18% to 30% in typically developing peers, driven by food selectivity, sensory aversions that restrict dietary variety, and gastrointestinal dysfunction (10–12).

Iron is essential for brain development, supporting myelination, dopamine synthesis, and neurotransmitter function; deficiency disrupts these processes and can produce cognitive, motor, and behavioral impairments that persist into adolescence, even after iron stores are repleted (13, 14). Consistent with this, a scoping review identified positive associations between iron-deficiency and neurodevelopmental disorders, including ASD (3). In typically developing children and those with attention-deficit/hyperactivity disorder, iron-deficiency is associated with greater behavioral difficulty, and supplementation has improved behavioral rating scale scores in controlled studies (15–17).

Despite this biological plausibility, few studies have examined the relationship between iron status and behavioral problems in ASD beyond reporting prevalence (12). Establishing whether such associations exist could inform screening and supplementation practices, a low-cost intervention with potential neurodevelopmental benefits (18, 19), and is particularly relevant in settings where anemia and ASD are both common (4, 9). Therefore, we examined the association between iron status and internalizing and externalizing behavioral problems in children newly diagnosed with ASD who were attending a tertiary care center in Western India. We hypothesized that ID and IDA would each be associated with higher odds of clinically significant behavioral problems than normal iron status.

## 2 Materials and methods

### 2.1 Study design and setting

This cross-sectional analytical study was conducted over 18 months (October 2023–March 2025) at the Child Development and Guidance Clinic (CDGC), Department of Pediatrics, a tertiary care medical college hospital in Western India. The study was approved by the Institutional Ethics Committee (BVDUMC/IEC/2022) and conducted in accordance with the Declaration of Helsinki and its subsequent amendments. Written informed consent was obtained from parents, with assent where appropriate, considering the children’s ASD status and developmental level. This study adhered to the STROBE guidelines.

### 2.2 Participants and data collection

The participants were children aged 2–18 years who were newly diagnosed with ASD according to the DSM-5 criteria. Eligibility was determined using consecutive sampling methods. The exclusion criteria were secondary ASD (e.g., associated with genetic syndromes or medical conditions), acute/chronic illnesses affecting iron metabolism (e.g., inflammatory bowel disease), hematologic disorders, and iron supplementation within three months prior to enrollment. Of the 210 screened children, 31 were excluded: four for secondary ASD, 25 for recent iron supplementation, and two because of caregiver refusal to provide informed consent. No child was excluded based on intercurrent illness or hematologic disorders. A total of 179 children were enrolled in this study.

### 2.3 Exposure and outcome definitions

#### 2.3.1 Autism diagnosis

ASD diagnosis was established using the DSM-5 criteria and confirmed using a standardized diagnostic instrument, the INCLEN Diagnostic Tool for ASD (INDT-ASD) (1, 20). The INDT-ASD was developed and validated among Indian children in accordance with the DSM-5 diagnostic criteria, exhibiting high sensitivity (97.3%) and specificity (98.4%) for ASD diagnosis across multisite Indian samples (20). It was specifically designed for application in resource-limited settings, where the accessibility and feasibility of administering internationally standardized instruments, such as the ADOS-2 or ADI-R, may be restricted. A developmental pediatrician confirmed all diagnoses.

#### 2.3.2 Behavioral assessment

Behavioral problems were assessed using parent-administered age-appropriate versions of the Child Behavior Checklist (CBCL/1.5–5 and CBCL/6–18) (21). Parents completed the CBCL before the disclosure of laboratory results, thus minimizing the risk of reporting bias based on their knowledge of their child’s iron status. The raw scores were converted to age- and sex-normalized T-scores. For our primary analysis, a T-score >63 on the internalizing or externalizing broad scales was considered clinically significant. A senior psychologist independently reviewed 10% of the CBCL T-score calculations and interpretations for accuracy.

#### 2.3.3 Iron status assessment

Hemoglobin levels were measured using an automated hematology analyzer (Beckman Coulter DxH 800/900), and serum ferritin levels were quantified using a chemiluminescence-based immunoassay. All assays were performed in a laboratory accredited by the National Accreditation Board for Testing and Calibration Laboratories (NABL) with daily internal quality control and external quality assurance. The interassay coefficients of variation were <2% for hemoglobin and <6% for ferritin. Laboratory personnel were blinded to the participants’ behavioral status. C-reactive protein and other inflammatory markers were not measured; the implications for possible misclassification of iron status are considered in the discussion.

Iron deficiency and iron-deficiency anemia were defined using age-specific WHO criteria (22, 23). In children aged <5 years, IDA was defined as serum ferritin <12 µg/L with hemoglobin <11.0 g/dL, whereas ID was defined as ferritin <12 µg/L with hemoglobin ≥11.0 g/dL. In children aged 5–11 years, IDA was defined as ferritin <15 µg/L with hemoglobin <11.5 g/dL, and ID as ferritin <15 µg/L with hemoglobin ≥11.5 g/dL. In children aged 12–18 years, IDA was defined as ferritin <15 µg/L with hemoglobin <12.0 g/dL, and ID as ferritin <15 µg/L with hemoglobin ≥12.0 g/dL.

For analysis, iron status was modeled as a single three-category variable — normal iron status, ID without anemia, and IDA — with normal iron status as the reference. A composite exposure comprising any ID or IDA was defined for secondary analyses.

### 2.4 Covariates

Covariates and demographic and socioeconomic data were collected using a structured questionnaire. The covariates included age in years as a continuous variable, sex, maternal education, and family structure. Maternal education was dichotomized as graduate or postgraduate versus below graduate level, following the NFHS-5 national survey categories (9), a distinction with implications for nutritional practices, anemia prevalence, and ASD severity. Family structure was categorized as nuclear (parents and children only) versus joint (multigenerational co-residential household), a prevalent arrangement in India with potential implications for dietary practices and caregiving. These covariates were selected based on their reported associations with iron status and behavioral outcomes (24).

### 2.5 Statistical analysis

#### 2.5.1 Data and variable definitions

Analyses were performed on a cohort of 173 children who had complete data on iron parameters and Child Behavior Checklist (CBCL) T-scores. Iron status was categorized into three distinct groups: normal iron status, iron-deficiency without anemia (ID), and iron-deficiency anemia (IDA), based on the criteria established by the World Health Organization (22, 23). Internalizing and externalizing behavior problems were each dichotomized at a CBCL T-score above 63, the established clinical cut-off.

#### 2.5.2 Sample size estimation

The sample size was determined *a priori* based on the longitudinal cohort study by Lozoff et al. (2000), which identified internalizing problems in 61% of children with a history of iron-deficiency, in contrast to 42% of children with sufficient iron levels (24). In the current study, the anticipated prevalence of internalizing problems was set at 75% for children with iron-deficiency and 50% for those with normal iron status for the *a priori* sample size determination. With a two-sided α of 0.05 and 80% power, 58 participants were required per group. Consecutive sampling yielded 173 participants in this study. No *post-hoc* power calculations were performed.

#### 2.5.3 Missing data

Six of the 179 enrolled children were excluded from the analysis owing to missing data on key variables: four with incomplete iron parameters and two with incomplete CBCL T-scores. The analytic sample was therefore 173 children, comprising 100 with normal iron status, 34 with ID, and 39 with IDA. All analyses were conducted using complete-case data; no imputation was performed.

#### 2.5.4 Descriptive statistics

Distributions of continuous variables were examined before analysis. Age and ferritin are presented as medians [interquartile range, IQR], whereas hemoglobin is presented descriptively as mean ± standard deviation (SD), with its 25th and 75th percentiles additionally reported to define the interquartile contrast. Categorical variables are presented as frequencies and percentages. Baseline characteristics were compared across the three mutually exclusive iron-status groups using Pearson’s chi-square test for categorical variables when all expected cell counts were at least five, Fisher’s exact test when any expected cell count was below five, and the Kruskal–Wallis test for age. Hemoglobin and ferritin were presented descriptively without formal comparison across iron-status groups because these biomarkers define the groups and any between-group differences are therefore inherent in the definitions. The overlapping Any ID/IDA column was presented descriptively and was excluded from significance testing.

#### 2.5.5 Analysis structure

The analyses were organized into three tiers for the revised analysis and presentation. The primary analysis examined the three-category iron-status variable against two co-primary outcomes, internalizing and externalizing problems. Secondary analyses examined the composite exposure comprising any ID or IDA and the continuous iron biomarkers. Alternative outcome thresholds, linear regression on continuous T-scores, within-stratum correlations, and model diagnostics were considered supportive or exploratory and are identified as such in the Results.

#### 2.5.6 Primary and secondary logistic models

Iron status was modeled as a single three-category variable with normal iron status as the reference, so that children with ID and children with IDA were each compared with iron-sufficient children rather than with one another. Crude odds ratios were estimated using univariable logistic regression. Multivariable logistic regression was adjusted for age in years as a continuous variable, sex, maternal education (graduate or postgraduate versus below graduate level), and family type (nuclear versus joint), yielding adjusted odds ratios with 95% confidence intervals. A global two-degree-of-freedom likelihood-ratio test was calculated for the three-category exposure within each outcome. Absolute prevalence differences between each exposure group and the reference group are reported alongside the odds ratios. The composite any ID/IDA exposure was evaluated in separate secondary models using the same reference group and covariate set; because the composite is a linear combination of the ID and IDA categories, it was never entered jointly with them. The linearity of the association between age and log odds was examined by adding a quadratic term.

#### 2.5.7 Multiple comparisons

Bonferroni correction was applied across the six adjusted exposure–outcome comparisons (α = 0.0083), using unrounded p-values. Although the composite exposure was specified as a secondary analysis, it was included in the correction family as a conservative approach. Results under Holm–Bonferroni, Benjamini–Hochberg false discovery rate, and outcome-specific Bonferroni procedures are provided in the **Supplementary Material**. Exploratory analyses were not corrected for multiplicity and are interpreted accordingly.

#### 2.5.8 Sparse-data and threshold sensitivity analyses

Because the ID and IDA strata were of modest size, models were examined for numerical signs suggestive of separation by assessing convergence, coefficient and standard-error magnitudes, and fitted probabilities. All maximum-likelihood models converged with finite estimates. Firth-type mean bias-reduced logistic regression was performed as a sparse-data sensitivity analysis for every primary and secondary model. Sensitivity analyses using alternative CBCL T-score thresholds of ≥60 and >65 were also performed.

#### 2.5.9 Continuous biomarkers and functional form

Ferritin and hemoglobin were examined as continuous variables using restricted cubic splines, which allow the association with behavioral problems to change across the biomarker range rather than assuming a constant effect per unit increase. A three-knot spline was used as the primary specification for all four biomarker–outcome combinations. During revision, this more parsimonious specification was chosen based on the sample size, the number of outcome events relative to the number of model parameters, and the instability of estimates from the more flexible four-knot models. Knots were placed at the 10th, 50th, and 90th percentiles of each biomarker’s observed distribution. Linear, natural-log-transformed, and four-knot models were also fitted and are reported in the **Supplementary Material**, compared using the Akaike information criterion together with likelihood-ratio tests for departure from linearity. Tests for nonlinearity are presented descriptively and were not used to select the primary model, and no multiplicity correction was applied to these model-form comparisons.

Ferritin and hemoglobin were evaluated in separate models and were never entered simultaneously. Each model was adjusted for age in years as a continuous variable, sex, maternal education (graduate/postgraduate versus below graduate level), and family type. For each biomarker, we report the overall likelihood-ratio test and adjusted odds ratios comparing clinically interpretable concentrations. These included the 25th versus 75th percentile and, for ferritin, increases across specified 5- and 10-µg/L intervals. This approach avoids implying that the same change in odds applies to every 1-µg/L increase across the entire biomarker range.

The tabulated odds ratios are conditional contrasts — comparisons of the fitted log odds at two specified biomarker concentrations. Because no biomarker-by-covariate interactions were included, these contrasts do not depend on the covariate values used to calculate them. The adjusted probability curves were obtained separately by averaging predicted probabilities over the observed covariate distribution, a procedure known as marginal standardization. Conditional odds ratios and marginal probabilities are not directly interchangeable because odds ratios are non-collapsible. Unadjusted associations between each biomarker and continuous CBCL T-scores were additionally summarized using Spearman’s rank correlation.

#### 2.5.10 Model performance

Model discrimination was quantified using the area under the receiver operating characteristic curve (AUC). For the three- and four-knot ferritin spline models, apparent AUCs were corrected for optimism using Harrell’s bootstrap procedure with 500 resamples; within each resample, spline knot positions were re-derived from the resampled biomarker distribution before refitting, and the refitted model was evaluated in both the resample and the original sample, with the mean difference being subtracted from the apparent AUC. Calibration was examined using the Hosmer–Lemeshow test based on ten predicted-risk groups; this test has limited power at the present sample size, and its results are reported without inference about calibration quality. Classification accuracy, calculated at a predicted-probability threshold of 0.50, is reported alongside the no-information rate, that is, the accuracy obtainable by assigning every child to the more frequent outcome category. These models were specified to estimate associations rather than predict individual outcomes, and the discrimination statistics are presented descriptively.

#### 2.5.11 Linear regression and exploratory analyses

CBCL internalizing and externalizing T-scores were additionally examined as continuous dependent variables using multivariable linear regression adjusted for the same covariates, yielding unstandardized β coefficients with 95% confidence intervals. Each predictor, the three-category iron-status variable, composite any ID/IDA exposure, and ferritin, was evaluated in a separate model; they were not entered simultaneously because the composite is a linear combination of the ID and IDA categories and ferritin enters the definition of both. Model assumptions were assessed using the Shapiro–Wilk test for residual normality, Breusch–Pagan test for homoscedasticity, variance inflation factors for multicollinearity, Cook’s distance for influence, and a quadratic term for the functional form of age. Inference was repeated using HC3 heteroscedasticity-consistent standard errors as a sensitivity analysis. Exploratory within-stratum associations between biomarkers and continuous T-scores were examined using Pearson and Spearman correlations within the ID and IDA strata separately.

### 2.6 Software and reproducibility

All analyses were performed in R version 4.5.0 (R Foundation for Statistical Computing, Vienna, Austria) using the base stats and splines packages version 4.5.0. Firth-type mean bias-reduced logistic regression was performed using the brglm2 version 1.1.0. HC3 robust standard errors, bootstrap optimism correction, and the Breusch–Pagan test were implemented in base R. Optimism-corrected areas under the receiver operating characteristic curve used 500 bootstrap resamples with spline knots re-estimated within each resample and random seed 20260730. Confidence intervals for the exploratory within-stratum correlations used 2,000 percentile bootstrap resamples with random seed 20260731. All tests were two-sided, and statistical significance was set at p < 0.05. Detailed package versions, knot arguments, random seeds, and estimation settings sufficient to reproduce the final model estimates, sensitivity analyses, and figures are provided in the **Supplementary Methods**.

## 3 Results

### 3.1 Study population

**Figure 1:**
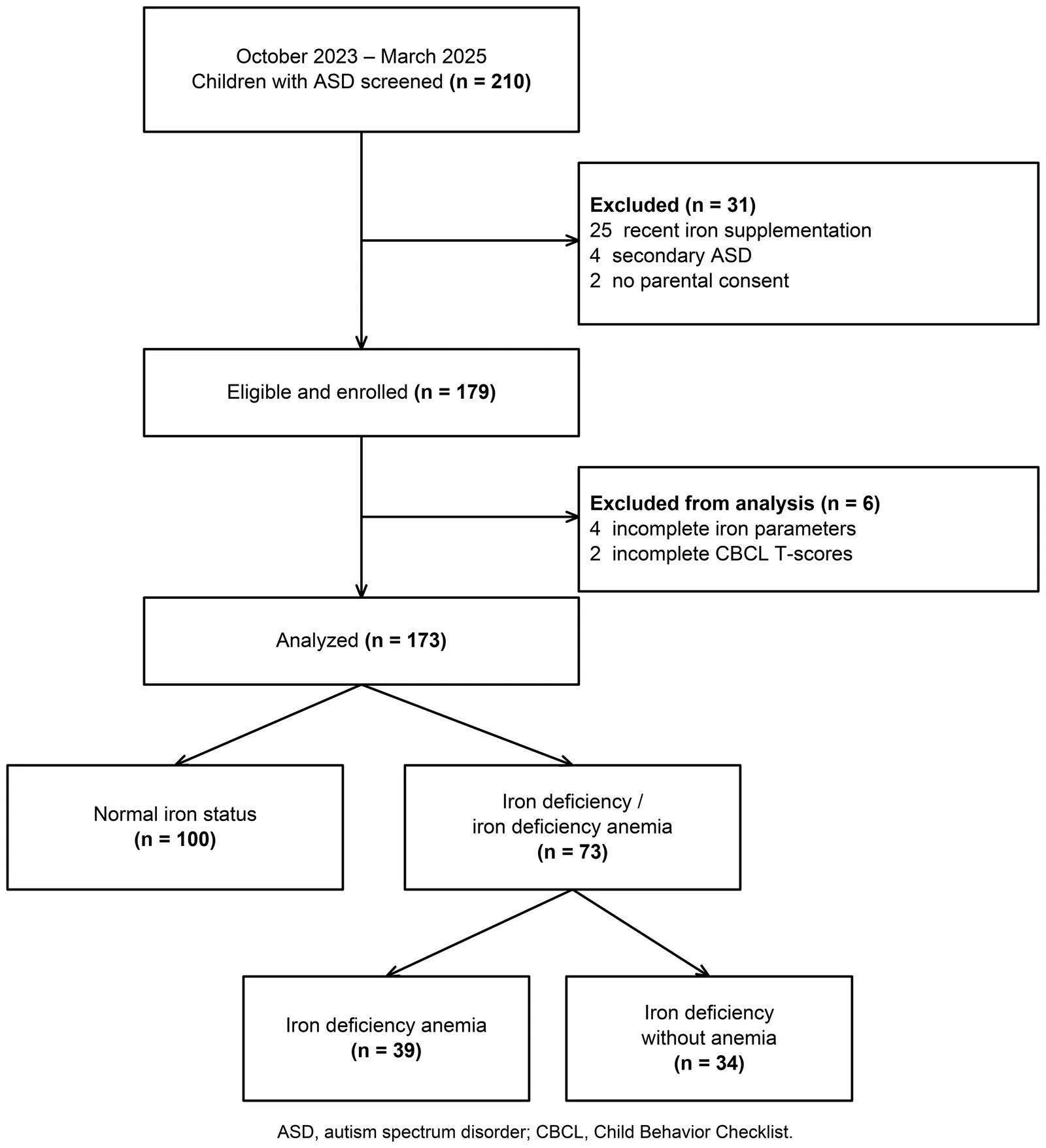
Participant flow diagram of the study. ASD, autism spectrum disorder; CBCL, Child Behavior Checklist. Of the 210 children with ASD screened (October 2023–March 2025), 31 were excluded at screening (25 with recent iron supplementation, 4 with secondary ASD, and 2 without parental consent), 179 were enrolled, and 173 were analyzed after excluding six with missing data. Of those analyzed, 100 had normal iron status and 73 had iron deficiency (ID, n=34) or iron deficiency anemia (IDA, n=39).

Of the 210 children screened, 31 were excluded (25 with iron supplementation within the preceding three months, four with secondary ASD, and two whose caregivers declined consent), and finally 179 were enrolled. Six were excluded from the analysis owing to missing data on key variables—four for incomplete iron parameters and two for incomplete CBCL T-scores—leaving 173 children for the analysis (**Figure 1**).

The median age of the cohort was 4.0 years [IQR 3.0–5.0]. Most children were male (138, 79.8%), had mothers educated to graduate level or above (116, 67.1%), and lived in nuclear families (163, 94.2%). Age differed across the three iron-status groups, being lower in the IDA group (median 3.0 years [2.0–4.0]) than in the normal iron-status and ID groups (4.0 years [IQR 3.0–5.0] in each; Kruskal–Wallis p = 0.012). Sex, maternal education, family type, and delivery mode did not differ significantly across groups (**Table 1**).

**Table 1:** Baseline characteristics by iron-status categories in children with autism spectrum disorder.

Variable	Category	Overall (173)	No ID/IDA (100)	ID (34)	IDA (39)	Either ID/IDA (73)	P value
Sex	M	138 (79.8)	77 (77.0)	27 (79.4)	34 (87.2)	61 (83.6)	0.406
	F	35 (20.2)	23 (23.0)	7 (20.6)	5 (12.8)	12 (16.4)	
Age, y, median [IQR]		4.0 [3.0-5.0]	4.0 [3.0-5.0]	4.0 [3.0-5.0]	3.0 [2.0-4.0]	4.0 [3.0-5.0]	0.012*^a^
Maternal education	Graduate/postgraduate	116 (67.1)	68 (68.0)	25 (73.5)	23 (59.0)	48 (65.8)	0.399
	Below graduate level	57 (32.9)	32 (32.0)	9 (26.5)	16 (41.0)	25 (34.2)	
Family type	Joint	10 (5.8)	7 (7.0)	1 (2.9)	2 (5.1)	3 (4.1)	0.902
	Nuclear	163 (94.2)	93 (93.0)	33 (97.1)	37 (94.9)	70 (95.9)	
Delivery mode	Vaginal	58 (33.5)	36 (36.0)	8 (23.5)	14 (35.9)	22 (30.1)	0.387
	LSCS	115 (66.5)	64 (64.0)	26 (76.5)	25 (64.1)	51 (69.9)	
Internalizing behavioral problem	No	104 (60.1)	71 (71.0)	15 (44.1)	18 (46.2)	33 (45.2)	0.003*
	Yes	69 (39.9)	29 (29.0)	19 (55.9)	21 (53.8)	40 (54.8)	
Externalizing behavioral problem	No	111 (64.2)	76 (76.0)	16 (47.1)	19 (48.7)	35 (47.9)	<0.001*
	Yes	62 (35.8)	24 (24.0)	18 (52.9)	20 (51.3)	38 (52.1)	
Hemoglobin^a^, g/dL,	mean [SD]	11.6 [1.2]	12.1 [0.8]	12.0 [0.7]	9.9 [0.8]	10.9 [1.3]	
Ferritin^a^, microgram/L, median [IQR]		12.4 [9.2–21.4]	18.7 [13.0–33.7]	7.8 [6.7–9.8]	7.8 [4.6–10.2]	7.8 [6.0–10.1]	

ASD, autism spectrum disorder; ID, iron deficiency without anemia; IDA, iron deficiency anemia; ID/IDA, presence of either ID or IDA; IQR, interquartile range; LSCS, lower-segment cesarean section; SD, standard deviation.

Values are presented as n (%), mean [SD], or median [IQR], as appropriate. P values compare the three mutually exclusive iron-status groups: normal iron status, ID, and IDA. The overlapping Either ID/IDA column is presented descriptively and was not included in the statistical comparisons.

^a^ Hemoglobin and ferritin distributions were determined by group definitions and are presented descriptively only; no formal comparison was made. The overall 25th and 75th percentiles of hemoglobin (11.0 and 12.4 g/dL) define the interquartile contrast reported in **Table 3**. Group-specific values were No ID/IDA 11.6 and 12.7, ID 11.5 and 12.6, and IDA 9.4 and 10.7 g/dL

Pearson’s chi-square test was used for categorical variables when the expected cell counts were at least 5. Fisher’s exact test was used for family type because of the small expected cell counts.^a^The Kruskal–Wallis test was used for age.

*Statistically significant at p < 0.05.

### 3.2 Prevalence of iron status and behavioral problems

Iron-deficiency without anemia was present in 34 children (19.7%), and iron-deficiency anemia was present in 39 (22.5%); 73 children (42.2%) had either. Internalizing problems were present in 69 children (39.9%) and externalizing problems were present in 62 children (35.8%). Both differed across the three iron-status groups: internalizing problems were present in 29.0% of children with normal iron status, 55.9% of those with ID, and 53.8% of those with IDA (p = 0.003), and externalizing problems in 24.0%, 52.9%, and 51.3%, respectively (p < 0.001). The median ferritin concentration was 18.7 µg/L [13.0–33.7] in children with normal iron status, 7.8 µg/L [6.7–9.8] in the ID group, and 7.8 µg/L [4.6–10.2] in the IDA group. These distributions were determined by the group definitions and were not formally compared (**Table 1**).

### 3.3 Association between iron status and behavioral problems

In the primary analysis, iron status was modeled as a single three-category variable with normal iron status as the reference. After adjustment for age in years, sex, maternal education, and family type, both ID and IDA were associated with higher odds of internalizing problems (ID: adjusted odds ratio [aOR] 3.03, 95% confidence interval [CI] 1.34–6.86, p = 0.008; IDA: aOR 2.73, 95% CI 1.23–6.05, p = 0.013) and externalizing problems (ID: aOR 3.71, 95% CI 1.63–8.46, p = 0.002; IDA: aOR 3.24, 95% CI 1.45–7.24, p = 0.004). The global two-degree-of-freedom likelihood-ratio test for iron status was significant for both outcomes (internalizing χ² = 10.56, p = 0.005; externalizing χ² = 14.19, p < 0.001).

Expressed as absolute differences, the prevalence of internalizing problems was 26.9 percentage points higher in the ID group and 24.8 points higher in the IDA group than in children with normal iron status; the corresponding differences for externalizing problems were 28.9 and 27.3 percentage points. In separate secondary models, any ID or IDA was associated with higher odds of internalizing problems (aOR 2.87, 95% CI 1.51–5.49, p = 0.001) and externalizing problems (aOR 3.46, 95% CI 1.79–6.70, p < 0.001) (**Table 2**).

**Table 2:** Association of internalizing and externalizing behavior problems in children with autism spectrum disorder by iron status.

Behavioral problems	Exposure	n/N	Prevalence difference^c^	Crude OR^a^ (95% CI)	Crude p-value	Adjusted OR^b^ (95% CI)	Adjusted p-value	Global adjusted p-value^d^
Panel A. Primary model: iron status as a single three-category variable (normal iron status as reference)								
Internalizing	ID	19/34	55.9% vs 29.0%; PD +26.9 pp	3.10 (1.39–6.92)	0.006^*^	3.03 (1.34–6.86)	0.008^*^	0.005
	IDA	21/39	53.8% vs 29.0%; PD +24.8 pp	2.86 (1.33–6.13)	0.007^*^	2.73 (1.23–6.05)	0.013^*^	
Externalizing	ID	18/34	52.9% vs 24.0%; PD +28.9 pp	3.56 (1.58–8.05)	0.002^*^	3.71 (1.63–8.46)	0.002^*^	<0.001
	IDA	20/39	51.3% vs 24.0%; PD +27.3 pp	3.33 (1.53–7.26)	0.002^*^	3.24 (1.45–7.24)	0.004*	
Panel B. Secondary model: composite Any ID/IDA variable (normal iron status as reference)								
Internalizing	Any ID/IDA	40/73	54.8% vs 29.0%; PD +25.8 pp	2.97 (1.58–5.58)	<0.001^*^	2.87 (1.51–5.49)	0.001^*^	—
Externalizing	Any ID/IDA	38/73	52.1% vs 24.0%; PD +28.1 pp	3.44 (1.80–6.58)	<0.001^*^	3.46 (1.79–6.70)	<0.001^*^	—

CI, confidence interval; ID, iron deficiency without anemia; IDA, iron deficiency anemia; OR, odds ratio; pp, percentage points; PD, prevalence difference.

In Panel A, ID and IDA were entered simultaneously as levels of a single three-category iron status variable, with normal iron status as the reference category. Panel B presents the composite Any ID/IDA exposure in separate secondary models.

^a^Crude ORs and p-values were estimated using univariable logistic regression.

^b^Adjusted ORs were estimated using multivariable logistic regression, controlling for age in years as a continuous variable, sex, maternal education (graduate/postgraduate vs. below-graduate level), and family type.

^c^Prevalence differences were compared between the exposure groups and children with normal iron status. Among the 100 children with normal iron status, 29 had internalizing problems and 24 had externalizing problems.

^d^Global p-values are from two-degree-of-freedom likelihood-ratio tests comparing the adjusted model containing the three-category iron-status variable with an otherwise identical covariate-only model. The corresponding likelihood-ratio statistics were χ² = 10.56 for internalizing problems and χ² = 14.19 for externalizing problems. The global tests evaluate ID and IDA jointly and do not represent either individual contrast. The global tests were not included in the six-comparison multiplicity-correction family.

^*^Statistically significant at p < 0.05.

Multiple-comparison analysis. Bonferroni correction was applied across the six adjusted exposure–outcome comparisons using unrounded p-values. Although the composite any ID/IDA exposure was specified as a secondary analysis, it was included in the correction family as a conservative approach. Five of the six associations remained statistically significant; the association between IDA and internalizing problems did not (Bonferroni-adjusted p = 0.079), and the association between ID and internalizing problems remained significant only narrowly (Bonferroni-adjusted p = 0.047). Results under alternative correction procedures are provided in **Supplementary Table 1**.

In the Firth-type sensitivity analysis (**Supplementary Table 4**), the ID–internalizing association also fell below the corrected threshold, indicating that this association was marginal in the sensitivity analysis.

### 3.4 Multiple comparisons

Bonferroni correction was applied across the six adjusted exposure–outcome comparisons using unrounded p-values. Five of the six associations remained statistically significant. The association between IDA and internalizing problems did not survive correction (adjusted p = 0.079), and the association between ID and internalizing problems survived only narrowly (adjusted p = 0.047); both should therefore be regarded as requiring confirmation in larger samples. Results under alternative correction procedures are presented in **Supplementary Table 1**.

### 3.5 Continuous iron biomarker associations

The assumption of a linear association between serum ferritin and the log odds of behavioral problems was formally assessed. A parsimonious three-knot spline was designated as the primary specification during revision based on the sample size, event counts, and estimate stability (**Supplementary Table 2**). For internalizing problems, there was clear evidence of departure from linearity (likelihood-ratio p < 0.001), whereas for externalizing problems, the three-knot spline did not indicate a departure from linearity (p = 0.227). Ferritin was associated with both outcomes in overall likelihood-ratio testing (both p < 0.001). An increase from the 25th percentile of ferritin (9.2 µg/L) to the 75th percentile (21.4 µg/L) was associated with lower odds of internalizing problems (aOR 0.23, 95% CI 0.11–0.46) and externalizing problems (aOR 0.30, 95% CI 0.15–0.60) (**Table 3A****).**

**Table 3:** Functional form and adjusted associations of serum ferritin and hemoglobin with behavioral problems in children with autism spectrum disorder (N = 173).

Panel A. Functional form and interquartile contrasts.						
Biomarker	Outcome (Behavioral problem)	Primary functional form	Overall association p^a^	Nonlinearity p^b^	Contrast (25th → 75th centile)	Adjusted OR (95% CI)^c^
Ferritin, µg/L	Internalizing	RCS, 3 knots	<0.001	<0.001	9.2 → 21.4	**0.23 (0.11–0.46)**
	Externalizing	RCS, 3 knots	<0.001	0.227^d^	9.2 → 21.4	**0.30 (0.15–0.60)**
Hemoglobin, g/dL	Internalizing	RCS, 3 knots	0.128	0.209	11.0 → 12.4	0.85 (0.54–1.32)
	Externalizing	RCS, 3 knots	0.040	0.018	11.0 → 12.4	1.10 (0.71–1.72)^e^
Panel B. Ferritin contrasts across clinically interpretable concentration ranges.						
Outcome	Ferritin contrast (µg/L)		Adjusted OR (95% CI)	p value	Change in strength across the range	
Internalizing	5 → 10		0.38 (0.23–0.60)	<0.001	Strongest association	
	10 → 15		0.46 (0.32–0.67)	<0.001		
	15 → 20		0.63 (0.50–0.78)	<0.001		
	20 → 30		0.77 (0.63–0.94)	0.012	Weakest association	
	**9.2 → 21.4 (interquartile)**		**0.23 (0.11–0.46)**	**<0.001**	**Summary contrast**	
Externalizing	5 → 10		0.52 (0.32–0.86)	0.010	Approximately uniform	
	10 → 15		0.57 (0.39–0.83)	0.003	across the range,	
	15 → 20		0.64 (0.51–0.81)	<0.001	consistent with the	
	20 → 30		0.55 (0.37–0.81)	0.002	nonlinearity test	
	**9.2 → 21.4 (interquartile)**		**0.30 (0.15–0.60)**	**<0.001**	**Summary contrast**	

CI, confidence interval; OR, odds ratio; RCS, restricted cubic spline.

All models were adjusted for age in years as a continuous variable, sex, maternal education (graduate/postgraduate vs. below graduate level), and family type. Each biomarker was evaluated in a separate model; ferritin and hemoglobin were not entered simultaneously. Knots for three-knot splines were placed at the 10th, 50th, and 90th percentiles of the observed distribution (ferritin: 5.91, 12.40, and 39.66 µg/L; hemoglobin: 9.90, 11.70, and 13.00 g/dL). Three knots were used for all four biomarker–outcome combinations, and the three-knot form was designated as primary during revision based on sample size, event count, and estimate stability.

^a^Likelihood ratio test comparing the full model with a model that omitted all biomarker terms.

^b^Likelihood ratio test comparing the spline specification with the corresponding linear specification.

**Figure 2:**
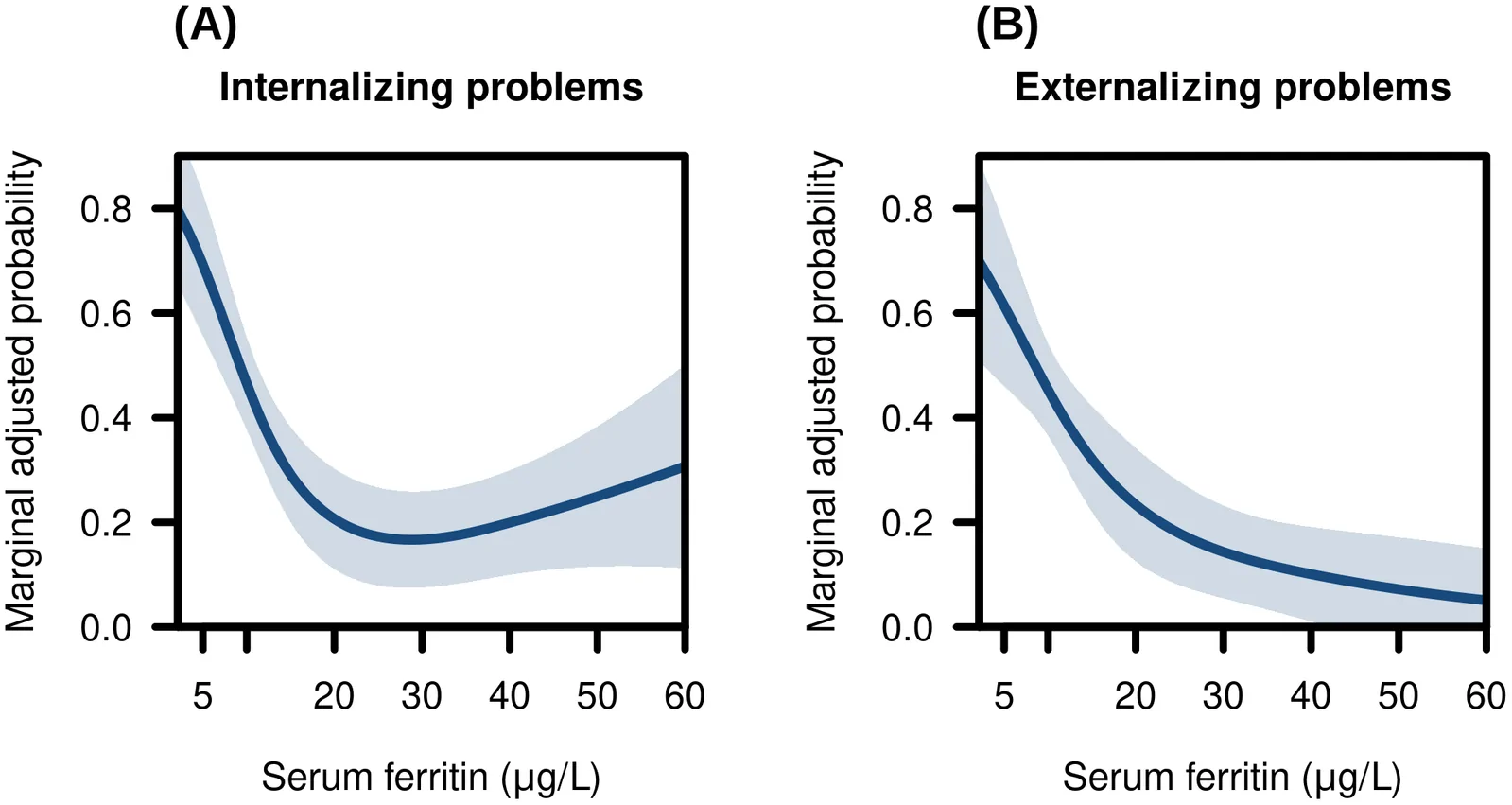
Adjusted associations between serum ferritin and behavioral problems in children with autism spectrum disorder (N = 173). Marginally standardized predicted probabilities of **(A)** internalizing and **(B)** externalizing problems across the displayed ferritin range were estimated using restricted cubic spline models with three knots at 5.91, 12.40, and 39.66 µg/L. Models were adjusted for age in years as a continuous variable, sex, maternal education (graduate/postgraduate versus below graduate level), and family type. Solid curves represent predicted probabilities standardized over the observed covariate distribution, and shaded regions represent 95% confidence bands. The x-axis is truncated at 60 µg/L; six children (3.5%) had ferritin concentrations above this value. All observations were retained in model estimation, but estimates in the sparsely populated upper range were correspondingly imprecise.

The tabulated odds ratios are conditional contrasts between two specified biomarker concentrations obtained from the fitted log odds. Because no biomarker-by-covariate interactions were included, these contrasts are invariant to the covariate values used to construct them. They do not represent a constant effect per unit increase and should be interpreted in conjunction with **Figure 2**. The adjusted probability curves in **Figure 2** were obtained by marginal standardization over the observed covariate distribution because odds ratios are non-collapsible; conditional contrasts tabulated here and marginal probabilities plotted in **Figure 2** are not directly interconvertible.

^d^For externalizing problems the three-knot spline did not indicate departure from linearity. The spline was retained because the same specification was applied to all four biomarker–outcome combinations rather than being selected on the basis of this test. In a four-knot sensitivity analysis, nonlinearity was statistically significant (p = 0.009), and the estimated shape differed materially; see **Supplementary Table 7** and Results.

^e^For hemoglobin and externalizing problems, the fitted association was non-monotonic and the interquartile contrast included the null. Taken with the non-significant unadjusted rank correlation (Spearman ρ = −0.067, p = 0.384), these analyses provide no reliable evidence of an association between hemoglobin and behavioral problems. Bold values indicate the primary interquartile contrast (75th versus 25th percentile of the observed ferritin distribution, 9.2 to 21.4 µg/L), which is the summary estimate reported in the text. The same contrast is shown in both panels.

A full model comparison across all 16 specifications is provided in **Supplementary Table 2**, discrimination statistics for the categorical models in **Supplementary Table 9**, optimism-corrected discrimination for the ferritin spline models in **Supplementary Table 6**, and four-knot sensitivity contrasts in **Supplementary Table 7**.

Interval-specific contrasts showed that the association was not uniform across the ferritin distribution for internalizing problems. It was strongest at the lowest concentrations and attenuated progressively as ferritin increased: adjusted odds ratios were 0.38 (95% CI 0.23–0.60) from 5 to 10 µg/L, 0.46 (0.32–0.67) from 10 to 15 µg/L, 0.63 (0.50–0.78) from 15 to 20 µg/L, and 0.77 (0.63–0.94) from 20 to 30 µg/L. For externalizing problems, the corresponding contrasts were approximately uniform across the range (0.52, 0.57, and 0.64 across successive 5 µg/L increments from 5 to 20 µg/L, and 0.55 from 20 to 30 µg/L), consistent with the non-significant test of nonlinearity for that outcome (**Table 3B**; **Figure 2**).

The apparent areas under the receiver operating characteristic curve for the three-knot ferritin models were 0.720 for internalizing and 0.718 for externalizing problems; after bootstrap optimism correction, these were 0.678 and 0.674, respectively. Although the interquartile contrasts were large, overall discrimination was modest. This is consistent with the shape of the association and the distribution of ferritin: the interquartile range spans the steepest segment of the fitted curve, whereas the upper tail of the distribution (90th percentile 39.7 µg/L, 95th percentile 55.5 µg/L) lies in the flatter region and contributes little to the discrimination between children with and without behavioral problems.

The same three-knot specification was applied to hemoglobin. There was no evidence of an overall association with internalizing problems (p = 0.128) or nonlinearity (p = 0.209), and the interquartile increase from 11.0 to 12.4 g/dL was not associated with internalizing problems (aOR 0.85, 95% CI 0.54–1.32). For externalizing problems, the spline showed evidence of nonlinearity (p = 0.018), and the overall association reached nominal significance (p = 0.040); however, the fitted association was non-monotonic, the interquartile contrast included the null (aOR 1.10, 95% CI 0.71–1.72), and the unadjusted rank correlation between hemoglobin and externalizing T-scores was not significant (Spearman ρ = −0.067, p = 0.384). Taken together, these analyses provide no consistent or reliable evidence of an association between hemoglobin concentration and behavioral problems in this cohort (**Supplementary Figure 1**).

### 3.6 Continuous behavioral outcomes

The CBCL T-scores were analyzed as continuous outcomes using separate adjusted linear regression models. Compared with normal iron status, both ID and IDA were associated with higher internalizing T-scores (ID: β 3.81, 95% CI 0.93 to 6.68, p = 0.010; IDA: β 3.67, 95% CI 0.87 to 6.47, p = 0.011). ID was associated with higher externalizing T-scores (β 5.04, 95% CI 1.69 to 8.40, p = 0.003), whereas the IDA estimate was smaller relative to its uncertainty and did not reach statistical significance (β 2.37, 95% CI −0.90 to 5.65, p = 0.154). In separate models, any ID or IDA was associated with higher internalizing (β 3.74, 95% CI 1.49 to 5.98, p = 0.001) and externalizing T-scores (β 3.66, 95% CI 1.03 to 6.30, p = 0.007). An increase from the 25th to the 75th percentile of ferritin was associated with lower internalizing T-scores (adjusted mean difference −4.53 points, 95% CI −6.69 to −2.37, p < 0.001) and lower externalizing T-scores (−4.63 points, 95% CI −7.15 to −2.11, p < 0.001) (**Table 4A**).

**Table 4:** Separate adjusted linear-regression models of iron status and serum ferritin with CBCL T-scores in children with autism spectrum disorder (N = 173).

Panel A. Adjusted mean differences in CBCL T-score.						
Outcome	Source model	Contrast		Adjusted β (95% CI)		p value
Internalizing	Three-category iron status	ID vs normal iron status		3.81 (0.93 to 6.68)		0.010*
		IDA vs normal iron status		3.67 (0.87 to 6.47)		0.011*
	Separate composite model	Any ID/IDA vs normal iron status		3.74 (1.49 to 5.98)		0.001*
	Separate ferritin RCS model	Ferritin 9.2 → 21.4 µg/L		−4.53 (−6.69 to −2.37)		<0.001*
Externalizing	Three-category iron status	ID vs normal iron status		5.04 (1.69 to 8.40)		0.003*
		IDA vs normal iron status		2.37 (−0.90 to 5.65)		0.154
	Separate composite model	Any ID/IDA vs normal iron status		3.66 (1.03 to 6.30)		0.007*
	Separate ferritin RCS model	Ferritin 9.2 → 21.4 µg/L		−4.63 (−7.15 to −2.11)		<0.001*
Panel B. Linear-regression assumption checks.						
Outcome	Model	Residual normality p	Homoscedasticity p	Maximum VIF	Maximum Cook’s D	Age nonlinearity p
Internalizing	Three-category iron status	0.226	0.852	1.14	0.072	0.456
	Separate composite model	0.235	0.799	1.04	0.060	0.472
	Separate ferritin RCS model	0.109	0.716	2.14	0.081	0.826
Externalizing	Three-category iron status	0.002	0.408	1.14	0.140	0.391
	Separate composite model	0.002	0.301	1.04	0.163	0.553
	Ferritin spline, three knots	<0.001	0.403	2.14	0.128	0.722

β, unstandardized adjusted mean difference in CBCL T-score; CBCL, Child Behavior Checklist; CI, confidence interval; ID, iron-deficiency without anemia; IDA, iron-deficiency anemia; VIF, variance inflation factor.

ID and IDA estimates were obtained from a single adjusted model containing iron status as a three-category variable, with normal iron status as the reference category. The composite any ID/IDA exposure was evaluated in a second, separate adjusted model and a ferritin RCS model in a third. Categorical iron status, composite exposure, and ferritin were never entered simultaneously because the composite is a linear combination of the ID and IDA categories, and ferritin enters the definition of both.

All models were adjusted for age in years as a continuous variable, sex, maternal education (graduate/postgraduate versus below graduate level), and family types. Ferritin was modeled using a restricted cubic spline with three knots, consistent with the specification used throughout; the tabulated ferritin estimate is the adjusted T-score difference comparing the 75th percentile (21.4 µg/L) with the 25th percentile (9.2 µg/L) and does not represent a constant difference per unit increase in ferritin.

The overall tests for the three-category iron-status variable were significant for internalizing (p = 0.005) and externalizing (p = 0.011) T-scores. The overall ferritin spline tests were significant for both outcomes (p <0.001).

Panel B: Residual normality was assessed using the Shapiro–Wilk test, homoscedasticity using the Breusch–Pagan test, multicollinearity using variance inflation factors, influence using Cook’s distance, and the functional form of age by adding a quadratic term. The maximum Cook’s distance across all models was 0.163. Residuals from the externalizing models departed from normality. Given the sample size, coefficient inference was expected to be reasonably robust asymptotically; HC3 heteroscedasticity-consistent standard errors were additionally used to reduce reliance on conventional model-based variance assumptions and produced materially unchanged estimates (**Supplementary Table 3**). Variance inflation factors for the spline models reflect the expected correlation between spline basis terms and do not indicate problematic collinearity among the covariates.

*Nominally significant at p <0.05.

Assumption checks showed no evidence of heteroscedasticity, problematic multicollinearity among the covariates, departure from linearity in age, or a single highly influential observation. The maximum Cook’s distance across all models was 0.163. The residuals from the externalizing models departed from normality. Given the sample size, coefficient inference was expected to be reasonably robust asymptotically; as an additional sensitivity analysis, HC3 robust standard errors were used to reduce reliance on conventional model-based variance assumptions, and the estimates and conclusions were materially unchanged (**Table 4B**; **Supplementary Table 3**).

### 3.7 Sensitivity and exploratory analyses

Firth-type mean bias-reduced logistic regression was performed as a sensitivity analysis because of the modest ID and IDA stratum sizes. The estimates were attenuated by approximately 3%–5% relative to the maximum-likelihood estimates but remained similar in direction and magnitude. All six associations were nominally significant; after Bonferroni correction, four remained significant—ID and IDA with externalizing problems and Any ID/IDA with both outcomes. The individual ID and IDA associations with internalizing problems did not survive correction, indicating greater uncertainty for these comparisons (**Supplementary Table 4**). Sensitivity analyses using alternative CBCL thresholds were generally but not uniformly confirmatory. Three of the four alternative-threshold analyses supported the primary associations, whereas the internalizing association at a T-score above 65 was directionally consistent but inconclusive (aOR 2.01, 95% CI 0.95–4.25, p = 0.066) (**Supplementary Table 5**).

In a four-knot sensitivity analysis of the ferritin association, the direction of effect was unchanged, but the estimated shape differed materially, and the optimism-corrected gain in discrimination attributable to the fourth knot was 0.009 for internalizing and 0.024 for externalizing problems (**Supplementary Tables 6**, **S7**). Therefore, the precise functional form should be regarded as sensitive to the spline complexity in a sample of this size. Exploratory within-stratum analyses detected no statistically significant correlations between either biomarker and CBCL T-scores within the ID or IDA strata; all confidence intervals included zero and were wide, reflecting small stratum sizes (**Supplementary Table 8**). The model discrimination and calibration statistics for the categorical models are presented in **Supplementary Table 9**.

## 4 Discussion

### 4.1 Principal findings

In this cross-sectional study involving 173 Indian children recently diagnosed with ASD, iron-deficiency and iron-deficiency anemia were prevalent, affecting 42.2% of the participants. After adjusting for age, sex, maternal education, and family type, it was found that children with iron-deficiency and those with iron-deficiency anemia had approximately three times higher odds of experiencing clinically significant behavioral problems compared with children with normal iron status. The global test for iron status was significant for both outcomes. In absolute terms, the prevalence of behavioral problems was 25 to 29 percentage points higher in the iron-deficient groups. Continuous biomarker analysis revealed an inverse association between serum ferritin concentration and behavioral problems, and analyses of continuous CBCL T-scores were consistent with the dichotomous models.

### 4.2 Prevalence and comparison with existing literature

The high prevalence of iron-deficiency observed in this study is consistent with data from ASD cohorts in low- and middle-income countries (10, 12). Food selectivity, sensory aversion, and gastrointestinal dysfunction restrict iron intake and absorption, and feeding difficulties characteristic of ASD compound this risk (11). Recent reviews have identified a high prevalence of micronutrient deficiencies in individuals with ASD across diverse populations (25). Studies on prevalence from high-resource settings report substantially lower figures: Reynolds et al. (26) found low serum ferritin in only 8% of US children with ASD, in contrast to the assumption that children with ASD are at universal risk of iron-deficiency. These discrepancies most likely reflect regional variations in background anemia rates, socioeconomic and dietary differences, and different diagnostic cutoffs.

The frequency of behavioral problems in this cohort (39.9% internalizing, 35.8% externalizing) is consistent with the elevated rates of co-occurring mental health difficulties documented in ASD (5–7). Adjusted odds ratios ranged from 2.73 to 3.71 across the categorical exposure–outcome comparisons. The direction of association was generally similar in supportive analyses, although precision and statistical support varied by outcome and analytic scale. These estimates are of a similar order to those reported in longitudinal work in typically developing children. Lozoff et al. reported that early iron-deficiency predicted behavioral difficulties over a decade later (24). However, as that study was prospective, its predictive framework is not applicable to the current cross-sectional data. The broader literature links low iron status to increased symptom severity in ADHD, with supplementation trials demonstrating improvements in behavioral rating scales (15–17). Few studies have examined iron status in relation to behavioral outcomes in ASD, and interventional evidence is limited. An RCT of ferrous sulfate in 20 children with ASD and low-normal ferritin (17–49 ng/mL) found no improvement in the primary sleep outcomes of sleep onset latency and wake time after sleep onset, and no between-group difference in daytime behavior assessed by the Aberrant Behavior Checklist, although iron status improved and the authors noted that interpretation was limited by low enrollment (27). Notably, that trial did not enroll children with confirmed iron-deficiency. Dosman et al. (28) found that iron supplementation improved ferritin levels and sleep in children with ASD, although they did not systematically evaluate behavioral outcomes. The current study is among the first to explore these associations in ASD using standardized behavioral assessments.

### 4.3 Mechanistic considerations

Iron functions as a cofactor for tyrosine and tryptophan hydroxylase, which are rate-limiting enzymes in the synthesis of dopamine and serotonin. Additionally, iron is essential for brain energy metabolism and myelination (13, 14). Iron-deficiency may exacerbate neurotransmitter systems that are already altered in ASD. Furthermore, neuroimaging data indicate reduced brain iron levels in children with ASD, with an inverse relationship observed between symptom severity and brain iron concentration (29). Mendelian randomization studies have also associated subcortical brain iron levels with the risk of ASD (30).

The analysis revealed that the association with internalizing problems was most pronounced at the lowest ferritin concentrations and progressively diminished as ferritin levels increased rather than following a uniform gradient. This pattern aligns with the saturating concentration–response relationship typical of micronutrients, where the functional consequences of deficiency are most significant when stores are severely depleted and decrease as sufficiency is approached. This observation is further supported by experimental evidence indicating that iron availability primarily limits monoamine synthesis and myelination under deficiency conditions rather than across the entire physiological range (31). The association with externalizing problems was relatively consistent across the observed range of ferritin, and formal testing did not reveal deviations from linearity.

### 4.4 Implications for clinical practice and public health

These findings support the incorporation of iron status screening and continued surveillance during the initial ASD evaluation. Iron-deficiency is among the most prevalent and clinically consequential nutritional deficiencies, and recent studies have emphasized the value of routine micronutrient screening in children with ASD (25). Given its established safety profile, iron replacement is a low-cost intervention that could complement existing behavioral and pharmacological management, although randomized controlled trials are required to establish whether correcting iron-deficiency improves behavioral outcomes. These implications are particularly relevant in low- and middle-income settings such as India, where childhood anemia exceeds 60% (8, 9) and ASD is underdiagnosed. In such contexts, addressing iron-deficiency through targeted supplementation or fortification may be a scalable strategy with broader child health benefits. Because behavioral problems in ASD substantially increase healthcare utilization and caregiver burden, the potential economic implications are considerable (6).

### 4.5 Strengths and limitations

The principal limitation of this study was its cross-sectional design, which precluded causal inference. We could not determine whether iron-deficiency contributes to behavioral problems, whether behavioral problems influence dietary intake and hence iron status, or whether both reflect a common underlying factor. Prospective and interventional studies are essential to clarify these relationships. The recruitment of participants from a single tertiary center, characterized by a cohort predominantly composed of males (79.8%), with a median age of 4.0 years, and originating from families with a high level of maternal education (67.1% holding a graduate degree or higher), may restrict the generalizability of the findings to community settings and ASD populations with differing characteristics.

Several potential confounders were not measured, including inflammatory markers, gastrointestinal symptoms, dietary intake, and other micronutrient deficiencies that commonly co-occur with iron-deficiency (25). Ferritin is an acute-phase reactant, and without inflammatory markers, we cannot exclude the misclassification of iron status. The severity of ASD was not characterized, which may independently affect both behavior and iron status. Standardized developmental assessments, such as cognitive, language, and motor developmental quotients, were not included in the study protocol. The developmental level may independently influence the presentation on the CBCL, potentially confounding or modifying the observed associations between iron status and behavioral outcomes in this study. Although the INDT-ASD is validated against DSM-5 criteria and appropriate for an LMIC setting, it was normed against the CARS rather than the ADOS-2 or ADI-R. The CBCL is parent-reported and, therefore, subject to reporting bias, although parents completed it before laboratory results were disclosed.

The *a priori* sample size calculation was based on a two-group comparison requiring 58 participants per group. The final analytic sample included 34 children with ID and 39 with IDA, both below the planned target. This reduced the precision of group-specific estimates, as reflected in the wide confidence intervals for the ID- and IDA-specific associations; the two internalizing associations did not meet the Bonferroni-corrected threshold and were positioned below it in the Firth-type sensitivity analysis, indicating they require confirmation in larger samples.

Two further analytical limitations merit mention. The estimated shape of the ferritin association was sensitive to spline flexibility: a three-knot model was designated as primary during revision based on sample size, event count, and estimate stability, and a four-knot alternative produced materially different interval-specific estimates while adding little to optimism-corrected discrimination. The overall inverse association was robust to this choice, but the precise functional form should be regarded as provisional. The categorical and continuous analyses of iron status are alternative parameterisations of the same exposure rather than independent lines of evidence, since serum ferritin defines the ID and IDA categories; their concordance reflects internal coherence rather than independent replication.

Dichotomous and continuous analyses were directionally consistent for every exposure, but the statistical support differed for IDA and externalizing problems. The adjusted odds ratio was significant (aOR 3.24, 95% CI 1.45–7.24, p = 0.004), whereas the corresponding linear coefficient was not (β 2.37, 95% CI −0.90 to 5.65, p = 0.154). The two analyses estimated distinct quantities: the odds ratio reflected the odds of crossing the clinical threshold of a T-score above 63, whereas the linear coefficient estimated the mean T-score difference across the full distribution, and a distributional shift concentrated near the threshold could appreciably change the proportion exceeding it while moving the mean comparatively little. The CI around the linear estimate was wide, reflecting imprecision within the IDA stratum (n = 39). No statistically significant mean difference was detected on the continuous scale, and this should not be interpreted as evidence that no such difference exists.

Despite these limitations, this study has several strengths. Iron status was determined using age-specific criteria established by the World Health Organization, and laboratory measurements were conducted in an accredited facility. Behavioral outcomes were evaluated using a standardized instrument completed prior to the disclosure of laboratory results. The analysis used a structured hierarchy of primary, secondary, supportive, and exploratory analyses, together with multiplicity correction, formal functional-form assessment, penalized-regression sensitivity analyses, and optimism-corrected discrimination estimates.

### 4.6 Future research directions

Prospective studies should incorporate the ADOS-2 or ADI-R to improve diagnostic precision and permit rigorous characterization of ASD severity and include validated developmental assessments such as the Vineland Adaptive Behavior Scales or the Bayley Scales to allow developmental-level-adjusted analysis. Randomized controlled trials employing standardized outcome measures are essential to ascertain whether iron supplementation enhances behavioral outcomes of children with ASD and iron-deficiency. Additionally, longitudinal studies monitoring iron status and behavior over time would clarify the temporal sequence and identify critical intervention windows. Larger sample sizes would enable adequately powered subgroup analyses, more precise characterization of the association across ferritin concentrations, and investigation of the interaction between iron-deficiency and other micronutrient deficiencies (25). The inclusion of inflammatory markers, dietary assessments, and objective behavioral measures would further elucidate these underlying mechanisms.

### 4.7 Conclusions

Iron-deficiency is common among Indian children with ASD and is linked to clinically significant behavioral problems. The associations were of similar magnitude across both ID and IDA, were biologically plausible, and demonstrated a graded relationship with serum ferritin concentrations. However, the association between IDA and internalizing problems did not survive correction for multiple comparisons, and the association between ID and internalizing problems survived only narrowly. These findings suggest that systematic screening for iron-deficiency can be considered as part of comprehensive care planning for children with ASD, particularly in comparable low- and middle-income settings where childhood anemia and ASD are both common. Randomized trials are necessary to determine whether correcting iron-deficiency improves behavioral outcomes.

## Data Availability

The raw data supporting the conclusions of this article will be made available by the authors, without undue reservation.
