# Supplementary material for: Association of iron deficiency and iron-deficiency anemia with behavioral problems in children with autism: a cross-sectional study
Source: Front Psychiatry. 2026 Sep 10;17:1900958. doi: 10.3389/fpsyt.2026.1900958 (PMC13601355; doi:10.3389/fpsyt.2026.1900958)
Supplement: Supplementary file 1 [file SupplementaryFile1.docx]

**SUPPLEMENTARY DIGITAL CONTENT**

Association of iron deficiency and iron-deficiency anemia with behavioral problems in children with autism: a cross-sectional study

Jatan Uday Merwana, MD , Vijay Raghunath Kalrao, MD, Ritika Chaudhry, MD, Leena Srivastava, MD

**TABLE OF CONTENTS**

| **Content Type** | **Description** | **Page Number** |
| --- | --- | --- |
| **Supplementary Methods** |  | **2–3** |
|  | S.1 Analytic sample and missing data | 2 |
|  | S.2 Variable coding | 3 |
|  | S.3 Knot placement | 4 |
|  | S.4 Estimands | 4 |
|  | S.5 Resampling procedures | 4 |
|  | S.6 Firth-type penalized regression | 4 |
|  | S.7 Multiplicity family | 5 |
|  | S.8 Sample size | 5 |
|  | S.9 Software | 5**–**6 |
|  | S.10 Data and code availability | 6 |
| **Supplementary Results** |  | 7 |
|  | S.R.1 Multiple-comparison procedures | 7 |
|  | S.R.2 Sparse-data and Firth-type sensitivity analysis | 7 |
|  | S.R.3 Model discrimination and calibration | 7 |
|  | S.R.4 Alternative outcome thresholds | 7 |
|  | S.R.5 Functional-form comparison | 8 |
|  | S.R.6 Optimism-corrected discrimination and functional-form sensitivity | 8 |
|  | S.R.7 Exploratory within-stratum correlations | 8 |
|  | S.R.8 Robust inference for the continuous outcomes | 9 |
| **Supplementary Tables** |  | 10 |
| **Supplementary Table S1** | Multiple-Comparison Corrections for Iron Status and Behavioral Outcome Associations | 10 |
| **Supplementary Table S2** | Comparison of functional-form specifications | 11 |
| **Supplementary Table S3** | HC3 robust sensitivity results | 12 |
| **Supplementary Table S4** | Sensitivity Analysis. Firth-Type Mean Bias-Reduced Logistic Regression | 13 |
| **Supplementary Table S5** | Sensitivity Analysis: Behavioral Outcomes at Alternative T-Score Cutoffs | 13 |
| **Supplementary Table S6** | Apparent and bootstrap optimism-corrected discrimination | 14 |
| **Supplementary Table S7** | Four-knot sensitivity analysis — ferritin contrasts | 14 |
| **Supplementary Table S8.** | Exploratory Within-Stratum Correlations of Iron Biomarkers With CBCL T-Scores in Children With Autism Spectrum Disorder | 15 |
| **Supplementary Table S9** | Logistic-Regression Model Diagnostics | 16 |
| **Supplementary Figure** |  |  |
| **Figure S1** | Adjusted associations between Hemoglobin concentration and Behavioral problems in children with autism spectrum disorder | 17 |
| **STROBE Checklist** |  | 18-24 |
| **References** |  | 24 |

## **Supplementary Methods. Detailed statistical specification**

### **S.1 Analytic sample and missing data**

Of 210 children screened between October 2023 and March 2025, 31 were excluded at screening (25 with recent iron supplementation, four with secondary ASD, and two without parental consent), and 179 were enrolled. Six were excluded from the analysis owing to missing data on key variables: four with incomplete iron parameters and two with incomplete CBCL T-scores. The analytic sample was therefore 173 children, comprising 100 with normal iron status, 34 with ID, and 39 with IDA. All analyses were complete-case analyses; no imputation was performed.

### **S.2 Variable coding**

| **Variable** | **Coding as analysed** |
| --- | --- |
| **Iron status** | Three-level factor: normal iron status (reference), ID without anemia, and IDA. For children aged <5 years, iron deficiency was defined as ferritin <12 µg/L; ID was ferritin <12 µg/L with hemoglobin ≥11.0 g/dL, and IDA was ferritin <12 µg/L with hemoglobin <11.0 g/dL. For children aged 5–11 years, iron deficiency was defined as ferritin <15 µg/L; ID was ferritin <15 µg/L with hemoglobin ≥11.5 g/dL, and IDA was ferritin <15 µg/L with hemoglobin <11.5 g/dL. For children aged 12–18 years, iron deficiency was defined as ferritin <15 µg/L; ID was ferritin <15 µg/L with hemoglobin ≥12.0 g/dL, and IDA was ferritin <15 µg/L with hemoglobin <12.0 g/dL. Ferritin thresholds were not adjusted for inflammation because inflammatory markers were not measured. |
| **Any ID/IDA** | Binary composite exposure: 0 = normal iron status (reference); 1 = ID or IDA. Analyzed in separate secondary models and never entered simultaneously with the three-category iron-status variable. |
| **Internalizing problem** | Binary: 1 = CBCL Internalizing T-score >63; 0 = T-score ≤63. There were 69 events. |
| **Externalizing problem** | Binary: 1 = CBCL Externalizing T-score >63; 0 = T-score ≤63. There were 62 events. |
| **Internalizing T-score** | Continuous CBCL T-score used as the dependent variable in supportive linear-regression analyses. |
| **Externalizing T-score** | Continuous CBCL T-score used as the dependent variable in supportive linear-regression analyses. |
| **Alternative CBCL thresholds** | Binary outcomes defined as a T-score ≥60 and T-score >65 for sensitivity analyses using the Any ID/IDA composite exposure. |
| **Age** | Continuous, in years. A quadratic age term was examined to assess departure from linearity. |
| **Sex** | Binary factor: female = reference; male = comparison category. |
| **Maternal education** | Binary factor: graduate or postgraduate = reference; below graduate level = comparison category. The three original categories were collapsed because the lowest category contained only seven children. |
| **Family type** | Binary factor: nuclear = reference; joint = comparison category. Ten children belonged to joint families. |
| **Hemoglobin** | Continuous, g/dL. Modelled using a restricted cubic spline with three knots at the 10th, 50th, and 90th percentiles—9.90, 11.70, and 13.00 g/dL—in biomarker analyses. Hemoglobin was evaluated in a separate model and was not entered simultaneously with ferritin or categorical iron status. |
| **Ferritin** | Continuous, µg/L. Modelled using a restricted cubic spline with three knots at the 10th, 50th, and 90th percentiles—5.91, 12.40, and 39.66 µg/L—in biomarker analyses. Ferritin was evaluated in a separate model and was not entered simultaneously with Hemoglobin or categorical iron status. |

### **S.3 Knot placement**

Knots were placed at the conventional percentiles of the observed distribution: the 10th, 50th, and 90th for three knots, and the 5th, 35th, 65th, and 95th for four knots. The ferritin knot values were 5.91, 12.40, and 39.66 µg/L (three knots) and 4.51, 10.54, 15.70, and 55.51 µg/L (four knots). The hemoglobin knot values were 9.90, 11.70, and 13.00 g/dL (three knots) and 9.30, 11.20, 12.10, and 13.14 g/dL (four knots). Contrasts were evaluated at the 25th and 75th percentiles of each biomarker (ferritin 9.16 and 21.4 µg/L; hemoglobin 11.0 and 12.4 g/dL) and for ferritin, additionally at 5, 10, 15, 20, and 30 µg/L.

### **S.4 Estimands**

Odds-ratio contrasts tabulated for the spline models are conditional contrasts of the fitted log odds, obtained as the difference in linear predictor between two biomarker values. Because the models contained no biomarker-by-covariate interaction terms, these contrasts do not depend on the covariate values at which they are evaluated. Confidence intervals for the marginally standardized probabilities were obtained using the delta method. The adjusted probability curves shown in **Figure 2** and **Supplementary Figure S1** are marginal; predicted probabilities were computed for every participant at each biomarker value and averaged over the observed covariate distribution. Because the odds ratio is non-collapsible, the conditional contrasts and marginal probabilities are not directly interconvertible, and an odds ratio derived from two points on the plotted curves will not equal the tabulated contrast.

### **S.5 Resampling procedures**

| **Procedure** | **Specification** |
| --- | --- |
| Bootstrap optimism correction of AUC | Optimism-corrected AUCs for the ferritin spline models were estimated using 500 bootstrap samples with random seed 20260730. Within each bootstrap sample, knot locations were re-derived from the resampled ferritin distribution before model refitting. Optimism was calculated as the difference between the AUC in the bootstrap sample and the AUC obtained by applying the bootstrap-fitted model to the original sample. The corrected AUC was the apparent AUC minus mean optimism. |
| Spearman confidence intervals | Spearman’s correlation confidence intervals were estimated using percentile bootstrap resampling with 2,000 samples and random seed 20260731. |

### **S.6 Firth-type Penalized regression**

Firth-type estimates were obtained using mean bias-reduced adjusted-score logistic regression with bias-reduced Wald confidence intervals. All standard maximum-likelihood models converged after four iterations with finite coefficients and standard errors, and fitted probabilities ranged from approximately 0.13 to 0.66, with no numerical signs suggestive of separation.

### **S.7 Multiplicity family**

The correction family comprised the six adjusted exposure–outcome comparisons: ID, IDA and any ID/IDA, each against internalizing and externalizing problems. The Bonferroni threshold was therefore α = 0.05 / 6 = 0.0083. Holm–Bonferroni and Benjamini–Hochberg procedures were applied across the same six comparisons; the outcome-specific Bonferroni procedure was applied within each family of three. All corrections used unrounded p-values.

### **S.8 Sample size**

The sample size was determined a priori from the longitudinal cohort study of Lozoff et al. (2000), which reported internalizing problems in 61% of children with a history of iron deficiency compared with 42% of iron-sufficient children. The sample size was determined a priori using an anticipated prevalence of internalizing problems of 75% among children with iron deficiency and 50% among children with normal iron status. With a two-sided α of 0.05 and 80% power, 58 participants were required per group. Consecutive sampling yielded 173 participants in this study. No post hoc power calculations were performed.

### **S.9 Software**

*Analytic environment.* All statistical analyses affected by the reviewer comments and all estimates reported in the final analysis tables were rerun using the final variable definitions, reference groups, covariates, and model specifications in R version 4.5.0 (R Foundation for Statistical Computing, Vienna, Austria).

*Core analyses.* Descriptive statistics, hypothesis tests, correlations, linear and logistic regression models, likelihood-ratio tests, model diagnostics, multiple-comparison corrections, and bootstrap procedures were implemented using the base R stats package version 4.5.0. Multiple-comparison adjustments used p.adjust() with the Bonferroni, Holm–Bonferroni, and Benjamini–Hochberg false discovery rate procedures, applied to unrounded model p-values.

*Spline modelling.* Natural restricted cubic splines were fitted using ns() from the splines package version 4.5.0. Because ns() distinguishes interior knots from boundary knots, the outermost specified percentiles were supplied as Boundary.knots and the remainder as interior knots. Therefore, three-knot models had boundary knots at the 10th and 90th percentiles of the observed biomarker distribution with one interior knot at the 50th percentile; four-knot sensitivity models had boundary knots at the 5th and 95th percentiles with interior knots at the 35th and 65th percentiles. Adjusted spline contrasts were obtained from the differences in the fitted model matrices between two specified biomarker values. Marginally standardized predicted probabilities and their delta-method confidence intervals were calculated by averaging model-based predictions over the observed covariate distribution.

*Penalized and robust estimation.* Firth-type mean bias-reduced logistic regression was fitted using brglmFit() with adjusted-score mean bias reduction from the brglm2 package version 1.1.0, with bias-reduced Wald confidence intervals (1). HC3 heteroscedasticity-consistent covariance matrices were calculated using a matrix implementation in base R rather than an additional robust-covariance package; HC3 inference was applied as a sensitivity analysis for the continuous CBCL outcomes.

*Discrimination and resampling.* The apparent areas under the receiver operating characteristic curve were calculated using the rank-sum definition of the area under the curve. Optimism-corrected values were estimated using a manual implementation of Harrell’s bootstrap procedure with 500 resamples and a random seed 20260730. Spline-knot locations were re-estimated within every bootstrap sample before the model was refitted, and the refitted model was evaluated in both the resample and the original analysis sample; mean optimism is the average difference between the two, subtracted from the apparent value. One of the 500 resamples for the three-knot externalizing model failed to converge and was excluded; all remaining resamples completed successfully. Confidence intervals for the Spearman correlations in the exploratory within-stratum analyses were estimated using percentile bootstrap resampling with 2,000 resamples and a random seed of 20260731.

*Diagnostics.* Regression assumptions were evaluated using residual diagnostics, the Shapiro–Wilk test, a manually implemented Breusch–Pagan test, variance inflation factors, Cook’s distance, leverage statistics, and quadratic terms for continuous-covariate functional for*m*

*Figures.* Figures were generated using the base R graphics and grDevices packages version 4.5.0 and exported as 300-dpi TIFF files at their final publication dimensions.

*Reproducibility materials.* All statistical tests were two-sided, and p < 0.05 was considered statistically significant. Multiplicity corrections were based on unrounded model p-values. All analyses used a complete-case sample of 173 children. The analysis code, random seeds, knot-placement rules, interval-contrast specifications, bootstrap outputs, prediction data, and publication figures were archived with the study reproducibility materials. For data handling and document preparation only, the study data workbook was checked and converted using Python version 3.13.13 with openpyxl version 3.1.5, and tables and reports were assembled using python-docx version 1.1.2; no statistical estimate reported in this manuscript was calculated in Python.

### **S.10 Data and code availability**

De-identified participant data and the study protocol may be available from the corresponding author ([vijay.kalrao@bharatividyapeeth.edu](mailto:vijay.kalrao@bharatividyapeeth.edu)) following publication, subject to Institutional Ethics Committee approval, and applicable consent and data protection requirements. The R code used to generate the reported estimates and figures is available from the corresponding author upon reasonable request

*.*

# **Supplementary Results**

### **S.R.1 Multiple-comparison procedures**

Under Bonferroni correction across the six adjusted exposure–outcome comparisons (α = 0.0083), five of six associations remained significant. The IDA–internalizing association did not (adjusted p = 0.079) and the ID–internalizing association survived narrowly (adjusted p = 0.047). Under the less conservative procedures all six associations remained significant: outcome-specific Bonferroni correction within families of three (adjusted p 0.004–0.040), the Benjamini–Hochberg false discovery rate procedure (0.001–0.013), and the Holm–Bonferroni step-down procedure (0.001–0.016). The conservative six-comparison Bonferroni result is the one reported in the main text (**Supplementary Table S1**).

### **S.R.2 Sparse-data and Firth-type sensitivity analysis**

All standard logistic regression models converged after four iterations with finite coefficients and standard errors, and fitted probabilities ranged from approximately 0.13 to 0.66, with no numerical signs suggestive of separation. Because the ID, IDA, and joint-family groups contained relatively few observations, the analyses were repeated using Firth-type mean bias-reduced logistic regression. Bias-reduced estimates were modestly attenuated but consistent in direction and magnitude with the primary estimates: ID and IDA remained associated with internalizing problems (ID aOR 2.91, 95% CI 1.29–6.56, p = 0.010; IDA aOR 2.64, 95% CI 1.19–5.82, p = 0.017) and with externalizing problems (ID aOR 3.52, 95% CI 1.55–8.00, p = 0.003; IDA aOR 3.11, 95% CI 1.39–6.92, p = 0.006), and the composite associations remained significant for both outcomes (internalizing aOR 2.77, 95% CI 1.46–5.28, p = 0.002; externalizing aOR 3.31, 95% CI 1.71–6.38, p < 0.001). After Bonferroni correction across the same six comparisons, four penalized associations remained significant — ID and IDA with externalizing problems, and the composite exposure with both outcomes — while the individual ID and IDA associations with internalizing problems did not meet the corrected threshold. This is consistent with the marginal status of the ID–internalizing association noted in the main analysis (**Supplementary Table S4**).

### **S.R.3 Model discrimination and calibration**

The categorical iron-status models showed modest discrimination, with areas under the receiver-operating-characteristic curve ranging from 0.669 to 0.680. All primary and secondary models improved on intercept-only models on likelihood-ratio testing (p = 0.006–0.017). The overall classification accuracy ranged from 62.4% to 67.6%, exceeding the no-information rate for both outcomes (60.1% for internalizing and 64.2% for externalizing problems). The Hosmer–Lemeshow p values ranged from 0.667 to 0.990; this test has limited power at the present sample size and these values are reported without inference about calibration quality. These models were specified to estimate associations rather than to predict individual outcomes, and the discrimination statistics are presented descriptively **(Supplementary Table S9).**

### **S.R.4 Alternative outcome thresholds**

Any ID or IDA was associated with internalizing problems at the lower threshold of a CBCL T-score of at least 60 (aOR 5.78, 95% CI 2.78–12.01, p < 0.001). At the more stringent threshold of a T-score above 65, the internalizing estimate remained elevated but was imprecise and did not reach statistical significance (aOR 2.01, 95% CI 0.95–4.25, p = 0.066). For externalizing problems, the association was significant at both the lower (aOR 4.06, 95% CI 2.08–7.93, p < 0.001) and the more stringent thresholds (aOR 2.88, 95% CI 1.37–6.07, p = 0.005). Three of the four alternative-threshold analyses therefore supported the primary associations, while the internalizing association at a T-score above 65 was directionally consistent but inconclusive. These sensitivity analyses used the composite exposure rather than the three-category variable to preserve precision at the more stringent threshold, where event counts are smaller (**Supplementary Table S5**).

### **S.R.5 Functional-form comparison**

For ferritin and internalizing problems, both spline specifications fitted better than the linear and log-transformed models (three knots: AIC 220.88, four knots 216.46, linear 233.96, log 227.21). For externalizing problems, the four-knot spline had the lowest AIC (208.94), whereas the three-knot spline did not improve the linear specification (214.94 versus 214.40), consistent with the non-significant test of nonlinearity for that outcome. For hemoglobin and internalizing problems, the linear (236.24) and log-transformed (235.89) specifications had marginally lower AIC than the three-knot spline (236.66); for externalizing problems, the three-knot spline had the lowest AIC (232.01). During revision, the parsimonious three-knot specification was designated as primary for all biomarker–outcome combinations based on sample size, event counts, events per parameter, and estimate stability; alternative forms were retained as sensitivity analyses (**Supplementary Table S2**).

### **S.R.6 Optimism-corrected discrimination and functional-form sensitivity**

The apparent areas under the curve for the three-knot ferritin models were 0.720 for internalizing and 0.718 for externalizing problems; mean bootstrap optimism was 0.041 and 0.044, respectively, giving corrected values of 0.678 and 0.674. The corresponding four-knot models gave corrected values of 0.687 and 0.698, a gain of 0.009 and 0.024, respectively. One of the 500 resamples for the three-knot externalizing model failed to converge and was excluded **(Supplementary Table S6).**

The two spline specifications implied different shapes. Under four knots, the association appeared absent below approximately 10 µg/L and steep above it (5 to 10 µg/L contrast: internalizing aOR 0.83, 95% CI 0.37–1.88; externalizing aOR 1.25, 95% CI 0.55–2.82), whereas under three knots it was strongest at the lowest concentrations and attenuated as ferritin increased. Interquartile contrasts differed 1.8-fold for internalizing and 2.7-fold for externalizing problems, and standard errors of the log odds ratio were 1.32 and 1.52 times larger under four knots **(Supplementary Table S7).**

### **S.R.7 Exploratory within-stratum correlations**

No statistically significant within-stratum associations were detected between ferritin and hemoglobin and CBCL T-scores. In the ID stratum, ferritin correlations with internalizing and externalizing T-scores were positive rather than inverse, although small and imprecise (Pearson r = 0.103 and 0.215, respectively). Hemoglobin was also positively correlated with externalizing T-scores in the ID stratum, but the confidence interval included zero (r = 0.326, 95% CI −0.013 to 0.598, p = 0.060). In the IDA stratum, ferritin correlations were close to zero, whereas hemoglobin correlations were negative; the largest estimate was for hemoglobin and internalizing T-scores (r = −0.309, 95% CI −0.569 to 0.007, p = 0.055). All confidence intervals included zero and were wide, reflecting substantial uncertainty in the small strata (ID n = 34, IDA n = 39). These exploratory results may reflect restricted biomarker ranges and sampling variability within strata and should not be interpreted as evidence of no association (**Supplementary Table S8**).

### **S.R.8 Robust inference for the continuous outcomes**

HC3 robust sensitivity analyses produced estimates consistent with the primary linear regression results. Compared with normal iron status, ID and IDA were associated with higher internalizing T-scores (ID: β 3.81, 95% CI 0.74–6.88, p = 0.015; IDA: β 3.67, 95% CI 0.95–6.39, p = 0.008), and the ferritin interquartile contrast was associated with lower internalizing T-scores (β −4.53, 95% CI −6.65 to −2.40, p < 0.001). For externalizing T-scores, ID remained associated (β 5.04, 95% CI 1.66 to 8.43, p = 0.004), and the IDA estimate remained non-significant (β 2.37, 95% CI −0.53 to 5.28, p = 0.109). Residuals from the externalizing models departed from normality. HC3 robust standard errors were used to reduce reliance on conventional model-based variance assumptions and produced materially unchanged estimates and conclusions, including for the ferritin interquartile contrast (β −4.63, 95% CI −7.29 to −1.98, p = 0.001) (**Supplementary Table S3**).

# **Supplementary Tables**

**Supplementary Table S1. Multiple-Comparison Corrections for Iron Status and Behavioral Outcome Associations**

| **Outcome** | **Comparison** | **Adjusted OR (95% CI)** | **Raw p** | **Bonferroni (six)** | **Bonferroni (within outcome)** | **FDR** | **Holm** |
| --- | --- | --- | --- | --- | --- | --- | --- |
| Internalizing | ID | 3.03 (1.34–6.86) | 0.008 | 0.047* | 0.023* | 0.009* | 0.016* |
|  | IDA | 2.73 (1.23–6.05) | 0.013 | 0.079 | 0.040* | 0.013* | 0.016* |
|  | Any ID/IDA | 2.87 (1.51–5.49) | 0.001 | 0.008* | 0.004* | 0.004* | 0.007* |
| Externalizing | ID | 3.71 (1.63–8.46) | 0.002 | 0.011* | 0.005* | 0.004* | 0.007* |
|  | IDA | 3.24 (1.45–7.24) | 0.004 | 0.025* | 0.012* | 0.006* | 0.012* |
|  | Any ID/IDA | 3.46 (1.79–6.70) | <0.001 | 0.001* | <0.001* | 0.001* | 0.001* |

Abbreviations: CI, confidence interval; FDR, false discovery rate; ID, iron deficiency without anemia; IDA, iron deficiency anemia; OR, odds ratio.

Adjusted odds ratios were obtained from the final logistic regression models, controlling for age in years as a continuous variable, sex, maternal education (graduate/postgraduate versus below graduate level), and family type. In the primary models, ID and IDA were entered together as levels of a single three-category iron-status variable, with normal iron status as the reference category. The composite any ID/IDA exposure was evaluated in separate secondary models using the same reference group.

Bonferroni (six) applies the correction across all six adjusted exposure–outcome comparisons and is the method reported in the main text. Bonferroni (within outcome) applies the correction separately within each outcome family of three comparisons. FDR values were calculated using the Benjamini–Hochberg procedure and Holm values using the Holm–Bonferroni step-down procedure, both across all six comparisons. All corrections used unrounded p-values. Although the composite exposure was specified as a secondary analysis, it was included in the correction family as a conservative approach.

* Statistically significant at p < 0.05 (adjusted).

**Supplementary Table S2. Comparison of functional-form specifications**

| **Outcome** | **Biomarker** | **Specification** | **AIC** | **ΔAIC** | **Nonlinearity p vs linear** |
| --- | --- | --- | --- | --- | --- |
| Internalizing | Ferritin | Linear | 233.96 | 17.50 | Reference |
|  |  | Natural log | 227.21 | 10.75 | Not applicable |
|  |  | **RCS, 3 knots (Primary)** | **220.88** | **4.42** | **<0.001** |
|  |  | RCS, 4 knots | 216.46 | 0.00 | <0.001 |
| Externalizing | Ferritin | Linear | 214.40 | 5.46 | Reference |
|  |  | Natural log | 215.94 | 7.00 | Not applicable |
|  |  | **RCS, 3 knots (Primary)** | **214.94** | **6.00** | **0.227** |
|  |  | RCS, 4 knots | 208.94 | 0.00 | 0.009 |
| Internalizing | Hemoglobin | Linear | 236.24 | 0.35 | Reference |
|  |  | Natural log | 235.89 | 0.00 | Not applicable |
|  |  | **RCS, 3 knots** **(Primary)** | 236.66 | 0.77 | 0.209 |
|  |  | RCS, 4 knots | 236.50 | 0.61 | 0.155 |
| Externalizing | Hemoglobin | Linear | 235.59 | 3.58 | Reference |
|  |  | Natural log | 235.23 | 3.22 | Not applicable |
|  |  | **RCS, 3 knots (Primary)** | **232.01** | **0.00** | **0.018** |
|  |  | RCS, 4 knots | 233.81 | 1.80 | 0.055 |

Abbreviations: AIC, Akaike information criterion; RCS, restricted cubic spline.

ΔAIC is calculated relative to the best-fitting specification within each biomarker–outcome combination. The specifications used in the primary analysis are shown in bold.

Restricted cubic splines with three knots were used for all four biomarker–outcome combinations, the three-knot form having been designated as primary during revision based on sample size, event count, and estimate stability; the supporting comparison of optimism-corrected discrimination is given in Table S6 and in the Results. Alternative specifications were fitted and are reported here for transparency but were not used to select the primary model. In two instances, the specification used did not have the lowest AIC: for ferritin and externalizing problems, the three-knot spline did not improve on the linear specification (214.94 versus 214.40), the four-knot spline having the lowest AIC (208.94); and for hemoglobin and internalizing problems, both the linear (236.24) and log-transformed (235.89) specifications had marginally lower AIC than the three-knot spline (236.66). The specified form was retained in both instances.

Knots were placed at conventional percentiles of the observed distribution: the 10th, 50th, and 90th for three knots, and the 5th, 35th, 65th, and 95th for four knots. The ferritin knot values were 5.91, 12.40, and 39.66 µg/L (three knots) and 4.51, 10.54, 15.70, and 55.51 µg/L (four knots). The hemoglobin knot values were 9.90, 11.70, and 13.00 g/dL (three knots) and 9.30, 11.20, 12.10, and 13.14 g/dL (four knots).

**Supplementary Table S3. HC3 robust sensitivity results**

| **Outcome** | **Contrast** | **HC3** β **(95% CI)** | **p value** |
| --- | --- | --- | --- |
| Internalizing | ID | 3.81 (0.74 to 6.88) | 0.015 |
|  | IDA | 3.67 (0.95 to 6.39) | 0.008 |
|  | Any ID/IDA | 3.74 (1.44 to 6.04) | 0.002 |
|  | Ferritin IQR contrast | −4.53 (−6.65 to −2.40) | <0.001 |
| Externalizing | ID | 5.04 (1.66 to 8.43) | 0.004 |
|  | IDA | 2.37 (−0.53 to 5.28) | 0.109 |
|  | Any ID/IDA | 3.66 (1.16 to 6.17) | 0.004 |
|  | Ferritin IQR contrast | −4.63 (−7.29 to −1.98) | 0.001 |

**Abbreviations:** β, unstandardized adjusted mean difference in Child Behavior Checklist T-score; CBCL, Child Behavior Checklist; CI, confidence interval; HC3, heteroscedasticity-consistent covariance estimator; ID, iron deficiency without anemia; IDA, iron deficiency anemia; ID/IDA, presence of either ID or IDA; IQR, interquartile range.

Estimates were obtained using ordinary least squares linear regression with HC3 robust standard errors (SEs) and confidence intervals (CIs). All models were adjusted for age in years as a continuous variable, sex, maternal education (graduate/postgraduate versus below graduate level), and family type. The ID and IDA estimates were obtained from one model containing iron status as a three-category variable, with normal iron status as the reference. Any ID/IDA and ferritin levels were evaluated in separate adjusted models and were not entered simultaneously with the three-category iron status variable. Ferritin was modeled using a three-knot restricted cubic spline; the ferritin IQR contrast represented the adjusted T-score difference comparing the 75th percentile (21.4 µg/L) with the 25th percentile (9.16 µg/L). HC3 inference was used as a sensitivity analysis to reduce reliance on conventional model-based standard errors. Statistical significance was set at p < 0.05.

**Supplementary Table S4. Sensitivity Analysis: Firth-Type Mean Bias-Reduced Logistic Regression**

| **Outcome** | **Model** | **Exposure** | **Standard MLE aOR (95% CI)** | **Firth-type aOR (95% CI)** | **Firth p- value** |
| --- | --- | --- | --- | --- | --- |
| Internalizing | Primary three-category | ID | 3.03 (1.34-6.86) | 2.91 (1.29-6.56) | 0.010 |
|  | Primary three-category | IDA | 2.73 (1.23-6.05) | 2.64 (1.19-5.82) | 0.017 |
|  | Secondary composite | Any ID/IDA | 2.87 (1.51-5.49) | 2.77 (1.46-5.28) | 0.002 |
| Externalizing | Primary three-category | ID | 3.71 (1.63-8.46) | 3.52 (1.55-8.00) | 0.003 |
|  | Primary three-category | IDA | 3.24 (1.45-7.24) | 3.11 (1.39-6.92) | 0.006 |
|  | Secondary composite | Any ID/IDA | 3.46 (1.79-6.70) | 3.31 (1.71-6.38) | <0.001 |

Abbreviations: aOR, adjusted odds ratio; CI, confidence interval; ID, iron deficiency without anemia; IDA, iron deficiency anemia; MLE, maximum-likelihood estimation. Models were adjusted for age in years as a continuous variable, sex, maternal education (graduate/postgraduate vs. below graduate level), and family type. Firth-type estimates were obtained using mean bias-reduced adjusted-score logistic regression; confidence intervals are bias-reduced Wald intervals. After Bonferroni correction across the same six comparisons (α = 0.0083), four of the six penalized associations remained statistically significant: ID and IDA with externalizing problems, and the composite exposure with both outcomes. The associations of ID and IDA with internalizing problems did not meet the corrected threshold (p = 0.010 and 0.017, respectively)

**Supplementary Table S5. Sensitivity Analysis: Behavioral Outcomes at Alternative T-Score Cutoffs**

| **Outcome** | **T-score cutoff** | **Any ID/IDA (n=73)** | **No ID/IDA (n=100)** | **Crude OR (95% CI)** | **Adjusted ORᵃ (95% CI)** |
| --- | --- | --- | --- | --- | --- |
| Internalizing problems | ≥60 | 59/73 | 44/100 | 5.36 (2.65-10.84) | 5.78 (2.78-12.01) |
|  | >65 | 21/73 | 17/100 | 1.97 (0.95-4.08) | 2.01 (0.95-4.25) |
| Externalizing problems | ≥60 | 54/73 | 41/100 | 4.09 (2.12-7.89) | 4.06 (2.08-7.93) |
|  | >65 | 25/73 | 15/100 | 2.95 (1.42-6.13) | 2.88 (1.37-6.07) |

Abbreviations: CI, confidence interval; ID, iron deficiency without anemia; IDA, iron deficiency anemia; OR, odds ratio. Reference group: children with normal iron status (no ID/IDA). The primary outcome definition was a CBCL T-score >63. Alternative cutoffs of ≥60 and >65 were evaluated as sensitivity analyses.

ᵃ Adjusted using multivariable logistic regression controlling for age in years as a continuous variable, sex, maternal education (graduate/postgraduate vs. below graduate level), and family type.

The adjusted p-values were <0.001 for internalizing T-scores at ≥60, 0.066 for internalizing at T-scores >65, <0.001 for externalizing at T-scores ≥60, and 0.005 for externalizing at T-scores >65.

Sensitivity analyses at alternative thresholds used the composite any ID/IDA exposure rather than the three-category iron-status variable to preserve precision at the more stringent cut-off where event counts are smaller. The composite comparison uses the same normal iron status reference group as the primary analysis.

**Supplementary Table S6. Apparent and bootstrap optimism-corrected discrimination**

| **Ferritin model** | **Outcome** | **Apparent AUC** | **Mean optimism** | **Corrected AUC** | **Valid resamples** |
| --- | --- | --- | --- | --- | --- |
| RCS, 3 knots (primary) | Internalizing | 0.720 | 0.041 | 0.678 | 500/500 |
| RCS, 3 knots (primary) | Externalizing | 0.718 | 0.044 | 0.674 | 499/500 |
| RCS, 4 knots (sensitivity) | Internalizing | 0.731 | 0.044 | 0.687 | 500/500 |
| RCS, 4 knots (sensitivity) | Externalizing | 0.745 | 0.047 | 0.698 | 500/500 |

**Abbreviations:** AUC, area under the receiver operating characteristic curve; RCS, restricted cubic spline. All models were adjusted for age in years, sex, maternal education, and family type. Apparent AUC was calculated in the original sample. Optimism was estimated using Harrell’s bootstrap procedure with 500 resamples drawn with replacement (random seed 20260730); spline knots were re-derived within each resample before model refitting, and each refitted model was evaluated in both the bootstrap and original samples. Mean optimism was the average difference between these AUCs, and corrected AUC = apparent AUC − mean optimism. One three-knot externalizing model failed to converge, leaving 499 valid resamples. The corrected AUC gain from the fourth knot was 0.009 for internalizing and 0.024 for externalizing problems*.*

**Supplementary Table S7. Four-knot sensitivity analysis — ferritin contrasts**

| **Outcome** | **Ferritin contrast (µg/L)** | **Four knots (sensitivity) aOR (95% CI), p** | **Three knots (primary): aOR (95% CI), p** |
| --- | --- | --- | --- |
| Internalizing | 5 → 10 | 0.83 (0.37–1.88), p = 0.663 | 0.38 (0.23–0.60), p < 0.001 |
|  | 10 → 15 | 0.40 (0.27–0.61), p < 0.001 | 0.46 (0.32–0.67), p < 0.001 |
|  | 15 → 20 | 0.41 (0.26–0.65), p < 0.001 | 0.63 (0.50–0.78), p < 0.001 |
|  | 20 → 30 | 0.42 (0.26–0.68), p < 0.001 | 0.77 (0.63–0.94), p = 0.012 |
|  | **9.2 → 21.4** | **0.13 (0.05–0.33), p < 0.001** | **0.23 (0.11–0.46), p < 0.001** |
| Externalizing | 5 → 10 | 1.25 (0.55–2.82), p = 0.596 | 0.52 (0.32–0.86), p = 0.010 |
|  | 10 → 15 | 0.40 (0.25–0.65), p < 0.001 | 0.57 (0.39–0.83), p = 0.003 |
|  | 15 → 20 | 0.35 (0.20–0.60), p < 0.001 | 0.64 (0.51–0.81), p < 0.001 |
|  | 20 → 30 | 0.30 (0.17–0.53), p < 0.001 | 0.55 (0.37–0.81), p = 0.002 |
|  | **9.2 → 21.4** | **0.11 (0.04–0.33), p < 0.001** | **0.30 (0.15–0.60), p < 0.001** |

**Abbreviations:** aOR, adjusted odds ratio; CI, confidence interval; IQR, interquartile range; RCS, restricted cubic spline. Each aOR compares the higher with the lower specified ferritin concentration and was adjusted for age in years, sex, maternal education, and family type. The 9.2→21.4 µg/L contrast represents the observed ferritin IQR. The three-knot model was the primary specification, and the four-knot model was a sensitivity analysis. The specifications produced different shapes: the four-knot association appeared weak below approximately 10 µg/L and steeper above it, whereas the three-knot association was strongest at the lowest concentrations and attenuated as ferritin increased. Four-knot versus three-knot IQR estimates differed 1.8-fold for internalizing and 2.7-fold for externalizing problems, with corresponding log-aOR standard errors 1.32 and 1.52 times larger. The detailed concentration–response shape should therefore be interpreted cautiously.

**Supplementary Table S8. Exploratory Within-Stratum Correlations of Iron Biomarkers With CBCL T-Scores in Children With Autism Spectrum Disorder**

| **Group** | **Biomarker** | **CBCL outcome** | **n** | **Pearson r (95% CI)** | **p value** | **Spearman rho (95% CI)** | **p value** |
| --- | --- | --- | --- | --- | --- | --- | --- |
| ID | Ferritin | Internalizing T-score | 34 | 0.103 (−0.243 to 0.427) | 0.561 | 0.061 (−0.279 to 0.405) | 0.733 |
|  |  | Externalizing T-score | 34 | 0.215 (−0.132 to 0.516) | 0.221 | 0.199 (−0.148 to 0.497) | 0.259 |
|  | Hemoglobin | Internalizing T-score | 34 | 0.079 (−0.266 to 0.407) | 0.655 | 0.028 (−0.320 to 0.367) | 0.874 |
|  |  | Externalizing T-score | 34 | 0.326 (−0.013 to 0.598) | 0.060 | 0.295 (−0.066 to 0.608) | 0.090 |
| IDA | Ferritin | Internalizing T-score | 39 | 0.027 (−0.291 to 0.339) | 0.871 | 0.026 (−0.288 to 0.348) | 0.876 |
|  |  | Externalizing T-score | 39 | 0.016 (−0.301 to 0.330) | 0.925 | −0.102 (−0.401 to 0.213) | 0.535 |
|  | Hemoglobin | Internalizing T-score | 39 | −0.309 (−0.569 to 0.007) | 0.055 | −0.219 (−0.499 to 0.133) | 0.180 |
|  |  | Externalizing T-score | 39 | −0.202 (−0.487 to 0.121) | 0.217 | −0.210 (−0.561 to 0.166) | 0.199 |

Abbreviations: ASD, autism spectrum disorder; CBCL, Child Behavior Checklist; CI, confidence interval; ID, iron deficiency without anemia; IDA, iron deficiency anemia; r, Pearson’s correlation coefficient; rho, Spearman’s rank correlation coefficient.

Both ferritin and hemoglobin levels were examined against internalizing and externalizing CBCL T-scores within each iron-status stratum. Pearson confidence intervals were obtained using Fisher ’s z transformation. Spearman confidence intervals were obtained using percentile bootstrap resampling with 2,000 resamples and random seed 20260731. P values are nominal and were not adjusted for multiplicity because the analyses were exploratory.

**Supplementary Table S9. Logistic-Regression Model Diagnostics**

| **Behavior problem** | **Model** | **AUC** | **HL p** | **LR p** | **% Correct** | **No-information rate** |
| --- | --- | --- | --- | --- | --- | --- |
| Internalizing | Primary three-category iron status | 0.679 | 0.667 | 0.011* | 63.0 | 60.1 |
|  | Secondary Any ID/IDA | 0.680 | 0.888 | 0.006* | 62.4 | 60.1 |
| Externalizing | Primary three-category iron status | 0.672 | 0.932 | 0.017* | 67.6 | 64.2 |
|  | Secondary Any ID/IDA | 0.669 | 0.990 | 0.009* | 67.1 | 64.2 |

Abbreviations: AUC, area under the receiver operating characteristic curve; HL p, Hosmer-Lemeshow goodness-of-fit p value; ID, iron deficiency without anemia; IDA, iron deficiency anemia; LR p, omnibus likelihood-ratio test p value; % Correct, overall classification accuracy.

Primary models included iron status as a single three-category variable: normal iron status, ID, and IDA, with normal iron status as the reference category. Secondary models evaluated the composite Any ID/IDA exposure separately. All models included age in years as a continuous variable, sex, maternal education (graduate/postgraduate vs. below graduate level), and family type.

Hosmer-Lemeshow tests were based on 10 predicted-risk groups. The classification accuracy was calculated using a predicted probability threshold of at least 0.50.

* Statistically significant at p < 0.05. No-information rate, the classification accuracy obtainable by assigning every participant to the more frequent outcome category

**Supplementary Figure S1**

**Figure S1. Adjusted associations between hemoglobin concentration and behavioral problems in children with autism spectrum disorder (N = 173).**

Marginally standardized predicted probabilities of **(A)** internalizing and **(B)** externalizing problems across the displayed hemoglobin range were estimated using restricted cubic spline models with three knots at 9.90, 11.70, and 13.00 g/dL. Models were adjusted for age in years as a continuous variable, sex, maternal education (graduate/postgraduate versus below graduate level), and family type. Solid curves represent predicted probabilities standardized over the observed covariate distribution, and shaded regions represent 95% confidence bands. The displayed x-axis begins at 9.0 g/dL; five children (2.9%) had hemoglobin concentrations below this value. All observations were retained in the model estimation. Neither the overall association nor the interquartile contrast was statistically significant for internalizing problems. For externalizing problems, the overall spline term reached nominal significance, but the fitted association was non-monotonic and the interquartile contrast included the null. The hemoglobin findings should therefore be interpreted cautiously.

# **STROBE Checklist ***

| **Section/Topic** | **Item** | **Recommendation** | **Where reported** | **Notes specific to this study** |
| --- | --- | --- | --- | --- |
| Title and Abstract | 1a | Indicate the study design in the title or abstract. | Title; Abstract | Cross-sectional analytical study of iron status and behavioral problems in children with ASD |
| Title and Abstract | 1b | Provide an informative and balanced summary in the abstract. | Abstract | Background, setting, N = 173 analyzed, exposure (three-category iron status: normal, ID, IDA), outcomes (CBCL internalizing/externalizing, T > 63), adjusted odds ratios with 95% CIs against a common normal-iron reference, prevalence differences, interquartile ferritin contrasts, and the result of Bonferroni correction (five of six associations retained). |
| Introduction | 2 | Explain the scientific background and rationale. | Introduction | Rationale linking iron deficiency to neurobehavioral outcomes in ASD, clinical significance, and evidence gap addressed. |
| Introduction | 3 | State specific objectives, including prespecified hypotheses. | Introduction; Methods 2.7 | For the revised analysis and presentation, the analyses were organized into three tiers. The primary analysis examined the three-category iron-status variable against the two co-primary behavioral outcomes. Secondary analyses examined the composite Any ID/IDA exposure and continuous iron biomarkers. Alternative outcome thresholds, continuous CBCL outcomes, within-stratum correlations, and model diagnostics were considered supportive or exploratory |
| Methods | 4 | Present the key elements of the study design early in the paper. | Methods 2.1 | Cross-sectional, clinic-based, analytical design. |
| Methods | 5 | Describe the setting, locations, and relevant dates. | Methods 2.1, 2.2 | Child Development and Guidance Clinic, tertiary care medical college hospital, Western India; October 2023 to March 2025; laboratory platforms for iron studies; CBCL administered before disclosure of laboratory results. |
| Methods | 6a | Give eligibility criteria and the sources and methods of participant selection. | Methods 2.2; Figure 1 | A total of 210 were screened, 31 were excluded at screening (25 with recent iron supplementation, 4 with secondary ASD, and 2 with no consent), 179 were enrolled, and 173 were analyzed after excluding six for missing data (four iron parameters and two [CBCL] T-scores). |
| Methods | 6b | For matched studies, give matching criteria and numbers. | Not applicable | No matching used. |
| Methods | 7 | Clearly define all outcomes, exposures, predictors, confounders, and effect modifiers. | Methods 2.3, 2.4, 2.7; Supplementary Methods S.2 | Exposure was modeled as a single three-category variable (normal iron status as reference, ID without anemia, and IDA), defined by age-specific WHO criteria. Under 5 years — IDA: ferritin < 12 µg/L AND Hb < 11.0 g/dL; ID: ferritin < 12 µg/L AND Hb ≥ 11.0 g/dL. 5–11 years — IDA: ferritin < 15 µg/L AND Hb < 11.5 g/dL; ID: ferritin < 15 µg/L AND Hb ≥ 11.5 g/dL. 12–18 years — IDA: ferritin < 15 µg/L AND Hb < 12.0 g/dL; ID: ferritin < 15 µg/L AND Hb ≥ 12.0 g/dL. Composite any ID/IDA was evaluated separately. Outcomes: CBCL internalizing and externalizing T-scores above 63. Covariates: age (continuous, years), sex, and maternal education (two levels) and family type. |
| Methods | 8 | For each variable, give data sources and details of assessment. | Methods 2.3; Supplementary Methods S.2 | CBCL/1.5–5 and CBCL/6–18, age- and sex-normalized T-scores. Hemoglobin by automated analyzer; ferritin by chemiluminescence immunoassay; NABL-accredited laboratory with internal and external quality assurance; interassay CV < 2% (Hb) and < 6% (ferritin). Laboratory staff blinded to the behavioral status. No inflammatory markers were measured; ferritin is an acute-phase reactant, and possible misclassification is acknowledged in the Discussion. |
| Methods | 9 | Describe any efforts to address potential sources of bias. | Methods 2.2, 2.6, 2.7; Discussion 4.5 | Selection: Participant flow and comparison of excluded versus analyzed participants. Information: standardized CBCL completed before laboratory disclosure, laboratory quality control, and blinded laboratory personnel. Confounding: multivariable adjustment with a single covariate set applied throughout. Sparse data: Models were examined for numerical signs of separation by assessing convergence, coefficient, and standard-error magnitudes, and fitted probabilities. All maximum-likelihood models converged with finite estimates. Firth-type mean bias-reduced logistic regression was performed as a sparse-data sensitivity analysis. Multiplicity: the primary multiplicity procedure selected for the revised analysis.. |
| Methods | 10 | Explain how the study size was arrived at. | Methods 2.5; Supplementary Methods S.8 | The sample size was determined a priori from Lozoff et al., who reported internalizing problems in 61% of children with a history of iron deficiency versus 42% of iron-sufficient children; assuming 75% versus 50% in ASD, a two-sided chi-square test with 80% power and 5% type I error required 58 per group. Consecutive sampling yielded 173 participants. No post-hoc power calculation was performed. |
| Methods | 11 | Explain how quantitative variables were handled in the analyses. | Methods 2.7; Supplementary Methods S.2, S.3 | CBCL outcomes were dichotomized at T > 63 for the primary analysis and analyzed as continuous T-scores in supportive linear models. Age was modeled continuously in years, with linearity assessed using a quadratic term. Ferritin and hemoglobin were modeled using restricted cubic splines using three knots at the 10th, 50th, and 90th percentiles of the observed distribution, the three-knot form being designated as primary during revision based on sample size, event count, and estimate stability. Maternal education was collapsed to two levels because the lowest of the three original categories contained seven children. |
| Methods | 12a | Describe all statistical methods, including those used to control for confounding. | Methods 2.7; Supplementary Methods | Univariable and multivariable logistic regression with a common reference category; global two-degree-of-freedom likelihood-ratio test for the three-category exposure; adjustment for age, sex, maternal education and family type. Bonferroni correction across the six adjusted exposure–outcome comparisons (α = 0.0083) reported in the main text; Holm–Bonferroni, Benjamini–Hochberg and outcome-specific Bonferroni results in Supplementary Table S1. |
| Methods | 12b | Describe any methods used to examine subgroups and interactions. | Methods 2.7; Supplementary Table S8 | Within-stratum correlations between iron biomarkers and continuous CBCL T-scores were examined separately in the ID and IDA strata. These analyses were exploratory, were not corrected for multiplicity, and were not designed for subgroup inference. |
| Methods | 12c | Explain how missing data were addressed. | Methods 2.6; Supplementary Methods S.1 | Complete-case analysis; no imputation. Six of 179 enrolled participants were excluded (4 missing iron parameters, 2 missing CBCL T-scores). The excluded and analyzed participants did not differ significantly on any measured baseline characteristic (all p > 0.05). |
| Methods | 12d | If applicable, describe analytical methods taking account of sampling strategy. | Methods 2.2 | Clinic-based convenience sample; no sampling weights were applied. |
| Methods | 12e | Describe any sensitivity analyses. | Methods 2.7; Supplementary Tables S3, S4, S5, and S7 | Firth-type mean bias-reduced logistic regression for all primary and secondary models; alternative CBCL thresholds (T ≥ 60 and T > 65); four-knot spline specification for ferritin; HC3 heteroscedasticity-consistent standard errors for the continuous outcome models; bootstrap optimism correction of model discrimination. |
| Results | 13a | Report numbers of individuals at each stage of study. | Results 3.1; Figure 1 | Participant flow is shown in Figure 1 and in the text. |
| Results | 13b | Give reasons for non-participation at each stage. | Results 3.1; Figure 1 | Screening exclusion and analysis exclusion were itemized with reasons. |
| Results | 13c | Consider use of a flow diagram. | Figure 1 | Flow diagram provided. |
| Results | 14a | Give characteristics of study participants. | Results 3.1; Table 1 | Age (median [IQR]), sex, maternal education, family type, and delivery mode; counts and percentages by iron-status group with group sizes in the column headers. |
| Results | 14b | Indicate number of participants with missing data for each variable of interest. | Methods 2.6; Results 3.1 | Six were excluded: four missing iron parameters and two missing CBCL T-scores. No missing data were found among the 173 patients analyzed. |
| Results | 14c | Summarize follow-up time if appropriate. | Not applicable | Cross-sectional design with no follow-up. |
| Results | 15 | Report numbers of outcome events or summary measures. | Results 3.2; Table 1 | ID 19.7% (n = 34), IDA 22.5% (n = 39), any ID/IDA 42.2% (n = 73); internalizing problems 39.9% (n = 69), externalizing problems 35.8% (n = 62); distribution by iron-status group in Table 1. |
| Results | 16a | Give unadjusted and adjusted estimates with precision. | Results 3.3, 3.5, 3.6; Tables 2, 3, 4 | Crude and adjusted odds ratios with 95% CIs, all against normal iron status as the common reference category, with adjusted p-values, and global likelihood-ratio tests (Table 2). The adjusted spline contrasts for continuous biomarkers (Table 3). The adjusted mean T-score differences were obtained from separate linear models (Table 4). The standardized effect sizes were not reported. |
| Results | 16b | Report category boundaries when continuous variables were categorized. | Methods 2.3, 2.7; Supplementary Methods S.3 | CBCL outcomes were dichotomized at T > 63, with alternative thresholds of T ≥ 60 and T > 65 in sensitivity analyses. Iron status was categorized using the age-specific WHO thresholds listed under item 7. Ferritin and hemoglobin were not categorized in the analysis; they were modeled continuously using restricted cubic splines with knots at ferritin 5.91, 12.40, and 39.66 µg/L and hemoglobin 9.90, 11.70, and 13.00 g/dL, and contrasts are reported between specified concentrations. |
| Results | 16c | Consider translating relative risk into absolute risk. | Results 3.3; Table 2 | Prevalence differences relative to normal iron status: internalizing problems +26.9 percentage points for ID and +24.8 for IDA; externalizing problems +28.9 for ID and +27.3 for IDA. For the composite exposure, +25.8 and +28.1 percentage points, respectively. Because the design is cross-sectional, these are prevalence differences rather than risk differences. |
| Results | 17 | Report other analyses done. | Results 3.4, 3.7; Supplementary Results; Supplementary Tables S1–S9 | Multiplicity corrections (S1); Firth-type sensitivity analysis (S4); model discrimination and calibration with the no-information rate (S9); alternative CBCL thresholds (S5), of which three of four supported the primary associations, while the internalizing analysis at T > 65 was directionally consistent but inconclusive; functional-form comparison (S2); optimism-corrected discrimination (S6); four-knot sensitivity contrasts (S7); exploratory within-stratum correlations (S8); and HC3 robust inference (S3). |
| Discussion | 18 | Summarize key results with reference to study objectives. | Discussion 4.1 | ID and IDA were each associated with approximately three-fold higher odds of internalizing and externalizing problems relative to normal iron status; prevalence differences of 25 to 29 percentage points; graded association between ferritin concentration and behavioral problems; continuous T-score analyses were directionally consistent. |
| Discussion | 19 | Discuss limitations. | Discussion 4.5 | Cross-sectional design precludes causal inference; single-center recruitment; unmeasured confounders including inflammatory markers, gastrointestinal symptoms, dietary intake, and other micronutrients; ASD severity and developmental level not characterized; INDT-ASD normed against CARS rather than ADOS-2 or ADI-R; parent-reported outcome measure; two of six associations attenuated after multiplicity correction; estimated concentration–response shape sensitive to spline complexity; categorical and continuous analyses represent the same exposure rather than independent evidence. |
| Discussion | 20 | Give a cautious overall interpretation. | Discussion 4.2, 4.3, 4.4, 4.7 | Findings contextualized against prior literature; association language used throughout; multiplicity results reported without overstatement; randomized trials identified as necessary to establish whether correcting deficiency improves outcomes. |
| Discussion | 21 | Discuss generalizability. | Discussion 4.4, 4.5 | Applicability to comparable clinic-based ASD populations in low- and middle-income settings; limitations noted for community samples and cohorts with different demographic profiles. |
| Other information | 22 | Funding. | Funding statement | No funding was received for conducting this study. |
| Other information | 23 | Ethical approval. | Ethics statement | Approved by the Institutional Ethics Committee (BVDUMC/IEC/2022); conducted in accordance with the Declaration of Helsinki; written informed consent was obtained from parents, with assent where developmentally appropriate. |
| Other information | 24 | Data availability and transparency. | Data availability statement; Supplementary Methods S.10 | De-identified participant data will be available upon reasonable request from the corresponding author following publication, subject to approval by the Institutional Ethics Committee of Bharati Vidyapeeth Medical College Hospital. The R code, random seeds, knot placement rules, and key numerical outputs were retained and are available from the corresponding author upon reasonable request. |

*Adapted from the STROBE Statement (2).

# **References**

1. Kosmidis I, Kenne Pagui EC, Sartori N. Mean and median bias reduction in generalized linear models. *Stat Comput*. 2020;30(1):43–59. doi:10.1007/s11222-019-09860-6
2. von Elm E, Altman DG, Egger M, Pocock SJ, Gøtzsche PC, Vandenbroucke JP; STROBE Initiative. The Strengthening the Reporting of Observational Studies in Epidemiology (STROBE) statement: guidelines for reporting observational studies. J Clin Epidemiol. 2008 Apr;61(4):344-9. doi: 10.1016/j.jclinepi.2007.11.008
